# Probing the Interactions of Porphyrins with Macromolecules Using NMR Spectroscopy Techniques

**DOI:** 10.3390/molecules26071942

**Published:** 2021-03-30

**Authors:** Ilche Gjuroski, Julien Furrer, Martina Vermathen

**Affiliations:** Department of Chemistry, Biochemistry and Pharmaceutical Sciences, University of Bern, Freiestrasse 3, 3012 Bern, Switzerland; ilche.gjuroski@dcb.unibe.ch (I.G.); julien.furrer@dcb.unibe.ch (J.F.)

**Keywords:** porphyrin, NMR spectroscopy, interaction, phospholipids, proteins, nucleic acids, drug delivery, polymer, cyclodextrin, surfactant, micelles

## Abstract

Porphyrinic compounds are widespread in nature and play key roles in biological processes such as oxygen transport in blood, enzymatic redox reactions or photosynthesis. In addition, both naturally derived as well as synthetic porphyrinic compounds are extensively explored for biomedical and technical applications such as photodynamic therapy (PDT) or photovoltaic systems, respectively. Their unique electronic structures and photophysical properties make this class of compounds so interesting for the multiple functions encountered. It is therefore not surprising that optical methods are typically the prevalent analytical tool applied in characterization and processes involving porphyrinic compounds. However, a wealth of complementary information can be obtained from NMR spectroscopic techniques. Based on the advantage of providing structural and dynamic information with atomic resolution simultaneously, NMR spectroscopy is a powerful method for studying molecular interactions between porphyrinic compounds and macromolecules. Such interactions are of special interest in medical applications of porphyrinic photosensitizers that are mostly combined with macromolecular carrier systems. The macromolecular surrounding typically stabilizes the encapsulated drug and may also modify its physical properties. Moreover, the interaction with macromolecular physiological components needs to be explored to understand and control mechanisms of action and therapeutic efficacy. This review focuses on such non-covalent interactions of porphyrinic drugs with synthetic polymers as well as with biomolecules such as phospholipids or proteins. A brief introduction into various NMR spectroscopic techniques is given including chemical shift perturbation methods, NOE enhancement spectroscopy, relaxation time measurements and diffusion-ordered spectroscopy. How these NMR tools are used to address porphyrin–macromolecule interactions with respect to their function in biomedical applications is the central point of the current review.

## 1. Introduction

**Figure 1:**
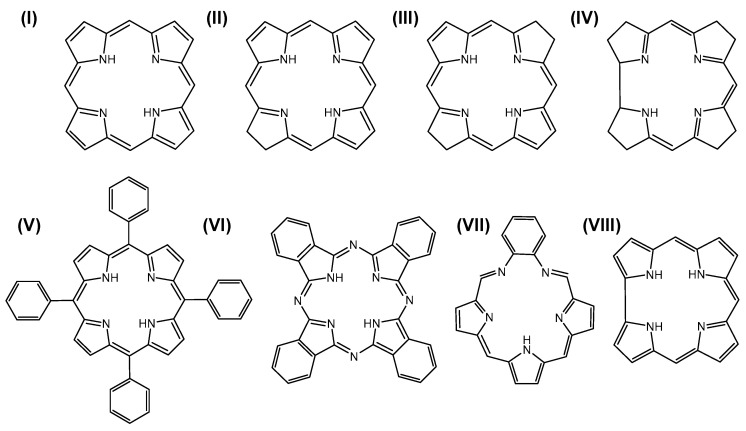
Structures of porphyrins (**I**), chlorins (**II**), bacteriochlorins (**III**), corrins (**IV**), tetraphenylporphyrins (**V**), phthalocyanines (**VI**), texaphyrins (**VII**), and corroles (**VIII**).

Porphyrinic compounds stand out among the organic molecules found in nature due to their unique properties associated with their common scaffold, a planar macrocycle consisting of four pyrrole rings linked by methine bridges [1]. The tetrapyrrole ring system forms a cavity that can accommodate many different metal ions, typically forming bidentate complexes that give rise to the widespread metalloporphyrins [2,3]. It is remarkable that a wide range of living systems including animals, plants and microorganisms make use of this common concept for fulfilling key functions in biological processes [4,5,6]. The major classes of naturally occurring porphyrinic compounds can be subdivided into porphyrins (**I**) in a narrower sense (often the whole group of porphyrinic compounds is referred to as porphyrins), chlorins (**II**), bacteriochlorins (**III**), and corrins (**IV**) (Figure 1) [3,4]. Heme forms the iron complex of protoporphyrin IX (PPIX) and its protein complex hemoglobin is the major constituent of red blood cells, imparting them their red color. The ability of hemoglobin and myoglobin to bind molecular oxygen plays an important role in oxygen transport and storage in living systems. Heme also functions as cofactor or prosthetic group in a vast number of hemoprotein-based enzymes such as the cytochromes that are involved in electron transfer and catalytic reactions [7,8]. Chlorins are partly reduced dihydro-porphyrins and their magnesium complexes form the core structure of chlorophylls, rendering the green color to plants [4]. The chlorophylls are part of the light-harvesting complexes of all photosynthetic organisms and thus fulfill one of the most important functions in life with their ability to use solar energy and transferring it to reaction centers so that molecular oxygen can be formed [9,10]. In bacteriochlorins, two opposite pyrrole rings are reduced, leading to tetrahydro-porphyrins (two adjacently reduced pyrrole rings yield isobacteriochlorins). They are part of bacteriochlorophylls in anoxygenic phototropic bacteria in the form of their Mg or Zn complexes [9,11]. Finally, the corrins have to be pointed out as modified tetrapyrroles whose Co(III) complexes forming vitamin B12 (cobalamin) are part of key metabolic enzymes [12].

The many different aspects of porphyrins such as synthesis, coordination chemistry, biochemistry, and applications have been compiled in a detailed compendium of numerous volumes devoted to this class of compounds, “The Porphyrin Handbook” [13]. Owing to their unique electronic structures, redox-, photochemical and photophysical properties, porphyrinic compounds have gained much interest in a broad range of applications in technology and biomedicine. Technical applications mainly rely on the capability of porphyrins for energy conversion, making them attractive materials for photovoltaic systems, photocatalysts and energy storage systems [14,15,16].

The most prevalent applications of porphyrinic compounds can be found in the biomedical field [17,18,19]. This is mainly due to the fact that porphyrins combine many advantageous properties such as light absorption in the visible and near-infrared wavelength region, intense fluorescence, ability to form toxic singlet oxygen from excited electronic triplet states following light irradiation (phototoxicity), preferential accumulation in tumor tissue, low dark toxicity, stability under physiologic conditions, and their ability to form complexes with various metal ions [20,21]. In addition, the tetrapyrrolic scaffold offers many possibilities for structural modifications, for example by introducing side chains onto the macrocycle, different metal ions into the core and expanding or reducing the ring system in order to tune the properties according to the desired features [22,23]. In addition to the naturally derived porphyrinic compounds given in Figure 1 (**I**–**IV**), the classes can be expanded towards modified synthetic derivatives such as the tetraphenylporphyrins (**V**) [24], phthalocyanines (**VI**) [25], porphyrins with extended ring systems such as texaphyrins (**VII**) [26], and corroles (**VIII**) [27] (Figure 1). Porphyrin-based drugs are used in both therapeutic and diagnostic areas [19]. For many decades now, the phototoxicity of porphyrins has been used in photodynamic therapy (PDT) of various oncologic and non-oncologic diseases [28] and PDT is the most important medical application of porphyrins to date. In PDT, the phototoxic reaction that leads to a selective tissue destruction is based on the formation of singlet oxygen and reactive oxygen species (ROS) via light excitation of the porphyrinic photosensitizer (PS) in the target tissue [21,29]. The same mechanism is also applied in the treatment of inflammations inactivating microorganisms in antimicrobial PDT [30,31]. Photodynamic diagnosis (PDD) measures the fluorescence of the PS enriched in tumor tissue or lesions [32,33,34]. Further imaging techniques make use of specific paramagnetic or radiolabeled metalloporphyrins as contrast agents for magnetic resonance imaging (MRI) [35,36], and positron emission tomography (PET) [37,38] or near-infrared (NIR)-absorbing porphyrins for photoacoustic imaging (PAI) [39,40] of tissue. Concomitant with the development of multimodal drugs, more recently, porphyrins are investigated as potential theranostics combining therapeutic and diagnostic functions [19,41,42]. Finally, the application of porphyrinic compounds in drug delivery should be mentioned in connection with the design of smart, stimuli-responsive platforms for controlled drug release. This includes for example photochemical internalization (PCI) [43,44] or porphyrin-based metal–organic frameworks (MOFs) [45]. Nanoplatforms have become an inevitable part in the application of porphyrinic drugs for solubilization, enhancing their efficiency and in vivo stability, and for passive and active tumor targeting [46,47,48,49]. Different strategies are applied in drug delivery of porphyrinic compounds, ranging from chemical binding to carrier or targeting molecules to physical entrapment into nanoparticles (NPs). Suitable NPs approved or under investigation consist of inorganic compounds [50], biodegradable carriers such as liposomes [51,52,53] or block copolymer micelles (BCMs) [54,55], molecular networks such as polyvinylpyrrolidone (PVP) [56,57] or cyclodextrins (CDs) [58,59] and numerous other materials [47,49]. In this vast range of biomedical applications, porphyrins are constantly exposed to macromolecules, either by the building blocks of their carrier materials or by the in vivo biological components such as plasma proteins or phospholipids in membranes. The interactions with these surrounding macromolecules form an important aspect as they often determine the stability, in vivo fate, efficiency and mechanisms of action of the porphyrinic agent. Analytical methods are therefore essential to address the different facets of interactions and nuclear magnetic resonance (NMR) spectroscopy can make an important contribution to this. To limit the scope of the current review, the focus lies on porphyrin derivatives investigated as medical drugs.

In general, most spectroscopic methods are well suited for porphyrin characterization. However, owing to their specific aromatic structure, porphyrins are predestined for spectrophotometric methods. The highly conjugated macrocyclic aromatic core of porphyrin molecules gives rise to strong light absorption with the characteristic Soret and Q-bands in the electron absorption spectra. From excited electronic states, radiative relaxation processes typically yield intense porphyrin fluorescence in the wavelength region of 620–700 nm [60]. Although the high sensitivity of the corresponding optical spectrometric instruments is beneficial for the analysis of porphyrin solutions at concentrations in the nano- and micro-molar range, at which most porphyrinic compounds exist in monomeric form, the structural and dynamic information important for comprehensive understanding of the interactions within the host–guest ensemble on a molecular or atomic level is rather limited.

NMR spectroscopy was discovered in 1946, and was introduced as a routine analytical tool in chemistry approximately 15 years later [61]. The first NMR investigations on porphyrin molecules were reported in 1959 [62] and since then the use of NMR for investigating porphyrin molecules steadily increased and continues to increase nowadays. Technical (hardware, computers) and methodological developments (democratization of 2D NMR spectroscopy and of pulsed field gradients (PFGs), new NMR pulse sequences) and technological breakthroughs (cryogenically cooled magnets and probeheads) have advanced NMR spectroscopy to a powerful analytical tool with increasing sensitivity and ability to address structural and dynamic questions in recent years. Therefore, these days, NMR spectroscopy is undoubtedly one of the most important analytical tool to study the structure of porphyrinic compounds and their metal complexes at the atomic level [63].

Our aim with this review is to provide the readers with an overview of several NMR techniques applied for characterizing *biomedical* porphyrinic compounds, focusing on their *interaction* with biological and synthetic macromolecules *in solution*. Essentially, these investigations are undertaken with the aim of getting deeper insights into the underlying mechanisms of interactions of these porphyrinic compounds. In particular, we wish to point out how NMR spectroscopic methods have contributed to the current knowledge in this field and why NMR spectroscopy is unique and complementary to other spectroscopic techniques. In Section 1, the most important NMR parameters and methods are briefly described with a short theoretical background. In Section 2, applications of these methods are presented in the context of porphyrins interacting with biomolecules such as proteins, phospholipids, or nucleic acids and with polymeric carrier systems. This overview is not exhausting but will rather point out the wide range of applications and the versatility of NMR spectroscopic methods in the field.

A list of abbreviations in alphabetical order is given in Appendix A at the end of this review.

## 2. NMR Parameters and Methods for Studying Porphyrinic Compounds and Their Surroundings

### 2.1. NMR Basics

NMR spectroscopy is a non-invasive technique for qualitative and quantitative analysis of a vast number of compounds. Similar to other spectroscopic methods, NMR spectroscopy finds its roots in the interactions between matter—specifically atomic nuclei with an active nuclear spin—and radiation—specifically electromagnetic fields. When a nuclear spin is placed into a strong static magnetic field (in an NMR spectrometer), the response of the spin polarization is to move around the field, at a specific precession rate, the Larmor frequency *ν*_0_. This Larmor frequency *ν*_0,_*_i_* of a given nucleus *i* depends on the external magnetic field *B*_0_ and the gyromagnetic ratio *γ_i_*, a nucleus-specific constant which correlates the magnetic moment of a nucleus to its angular momentum (Equation (1)). If we consider a sample with *N* nuclear spins, a stable anisotropic distribution of nuclear spin polarizations gradually takes place (the buildup and decay of longitudinal spin magnetization follows an exponential process, governed by *T*_1_, the longitudinal relaxation time) and leads to a small net magnetic longitudinal moment (*M_z_*) along the field *B*_0_ (*z*-direction). Since this longitudinal nuclear spin magnetization is nearly undetectable, the magnetization perpendicular *(M_x_ or M_y_)* to the external field is measured. A perpendicular magnetization is obtained by applying a radiofrequency (RF) pulse of appropriate frequency *ν*_1_, equal to the Larmor frequency *ν*_0_ of the nuclei that one intends to observe, and of appropriate duration [64].

(1) $$\nu_{0,i}=\frac{\gamma_{i}}{2\pi }B_{0}$$

One of the strengths of NMR spectroscopy is that the Larmor frequency *ν*_0,_*_i_* of a given nucleus *i* or its chemical shift *δ_i_* does not actually depend on the external magnetic field *B*_0_, but on the effective field *B_eff_*, which is the external magnetic field *B*_0_ corrected by a contribution *σ_i_* (shielding constant), a measure of the shielding of the nucleus *i* originating from the movement of the surrounding electrons (Equations (2) and (3)).

(2) $$B_{eff,i}=\;B_{0}(1\;-\;\sigma_{i})$$

(3) $$\nu_{0,i}=\frac{\gamma_{i}}{2\pi }B_{0}\left(1-\sigma_{i}\right)$$

As such, for a given molecule, the different shielding constants *σ_i_* and thus the different Larmor frequencies *ν*_0,_*_i_* are detectable by the NMR spectrometer [65,66] and give rise to NMR spectra in which *all* non-equivalent nuclei appear with distinguishable resonance frequencies.

In practice, the measurement and labeling of the absolute frequencies have rapidly appeared as unpractical, since they depend on the external magnetic field *B*_0_. Therefore, a new, *B*_0_-independent parameter, the chemical shift *δ*, was introduced. Indeed, the chemical shift *δ* is the relative deviation (offset) from the reference (onset) frequency with respect to the reference frequency, given in ppm (Equation (4)). In proton and carbon spectra, the chemical shift values are referenced to the resonance of tetramethylsilane (TMS, *δ* = 0 ppm) for organic solvents or alternatively of 2,2-dimethyl-2-silapentane-5-sulfonate (DSS, *δ* (Trimethylsilyl) = 0 ppm) for aqueous solutions.

(4) $$\delta_{i}=\frac{\nu_{i}\left(sample\right)-\nu_{0}\left(reference\right)}{\nu_{0}\left(reference\right)}$$

In addition to the chemical shift, the scalar-scalar or indirect coupling *J* and the direct or through-space coupling (Nuclear Overhauser Effect, NOE, see Section 2.3) between nuclear spins provide additional valuable information on the chemical environment of a given nucleus. Specifically, the *J*-coupling splits the resonance (multiplicity) of the observed nucleus according to the number of NMR active nuclei present in the direct vicinity (commonly up to five chemical bonds, but possible up to seven bonds). Additionally, the value of the coupling constant *J* can provide useful information about the structure and the stereochemistry of the investigated molecule. The most useful coupling constants are undoubtedly the vicinal (^3^*J*) coupling constants. Their values are dependent on the dihedral angle Θ, and for protons in a typical H_1_-X-X-H_2_ fragment, range from ~0 Hz (Θ ~ 90°) to ~15 Hz (Θ ~ 0° or 180°), following the Karplus equation [67,68,69]. This relationship is valuable for determining the stereochemistry of molecules, particularly of sugars, and for determining the backbone torsion angles in protein NMR studies.

Thus, relying on the NMR phenomena important parameters can be obtained for each NMR active nucleus (spin quantum number ≠ 0) within a molecule such as the chemical shift *δ* (a normalized measurement of the Larmor frequency *ν*_0_, independent of the external magnetic field *B*_0_), the scalar coupling *J* or through space spin polarization transfer (NOE).

### 2.2. Induced Spectral Perturbation

#### 2.2.1. The Porphyrin Ring Current Effect

The overall shielding constant *σ* for each group described in Equation (3) is formed as the *sum* of the following contributions (Equation (5)):

*σ* = *σ_dia_* + *σ_para_* + *σ_R_* + *σ_ext_* (5)

**Figure 2:**
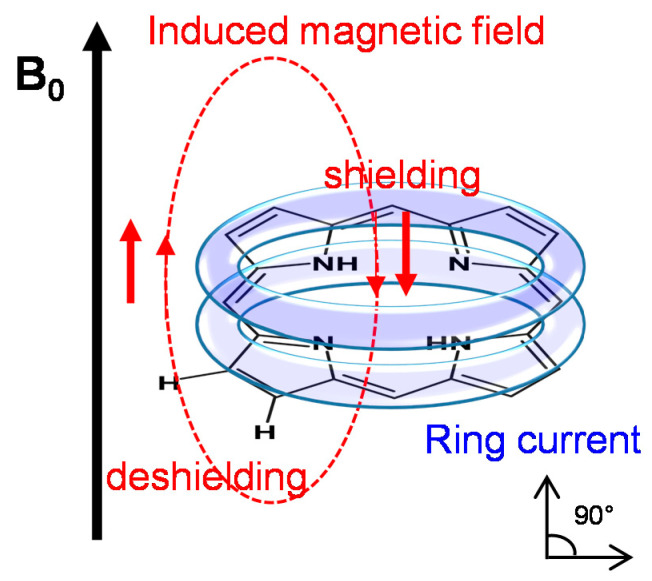
Scheme of porphyrin ring current.

The diamagnetic contribution *σ_dia_* derives from the electron distribution in spherically symmetrical orbitals surrounding the nucleus (*s* orbitals). The paramagnetic shielding term *σ_para_* arises from magnetic fields generated by non-spherically distributed electrons. It originates from excited electronic states that appear upon interaction of the electron orbitals with the applied magnetic field and generally becomes more important for other non-proton nuclei like, e.g., in ^13^C-NMR [65,66]. Of particular interest for NMR spectra of porphyrins and interacting molecules are the ring current term *σ_R_* and the external (intermolecular) shielding term *σ_ext_* derived from interactions with the neighboring molecules. Local magnetic anisotropy and unusual chemical shifts for the nuclei in the porphyrin core are well described by the ring current model. Mainly, protons inside (in the center) or above the porphyrin ring are in the shielding region while the protons placed in the porphyrin plane periphery are in the deshielding region of the ring current effect (Figure 2) [70]. These interactions, which are diamagnetic in origin, are generated by the neighboring groups in the vicinity such as the delocalized π-systems or the carbonyl groups and depending on the orientation and distance regarding the observed core can produce shielding or deshielding contributions [70].

This concept of ring current effect was introduced by Pauling, elaborating the appearance of anisotropic magnetic susceptibility of the benzene core, ascribing the magnetic anisotropy to the ring current effect, i.e., to induced circulation of the delocalized π- electrons by the magnetic field [71]. Further, empirical description and application of the ring current model for the NMR calculations (chemical shift) were reported by Pople, explaining the unusual chemical shift of the cyclic aromatic molecules compared to the alkanes [72]. Later, the shortfalls of the Pople (dipolar) model were overcome with the ring current models reported by Fassenden and Waugh [73,74] and Johnson and Bovey [75], modelled in terms of electron flow in loops located above and below the aromatic plane. Recent models use either five-loop models (four for the pyrrole ring and one for the macrocycle) or eight-loop models (the four pyrrole rings and the four hexagons between the central metal atom, neighboring pyrrole atoms and the meso position). Current models are approximations of either the current loop model of Johnson and Bovey [75] or the dipole model developed by Abraham et al. [76,77,78] and they are in good agreement to the observed NMR shifts.

#### 2.2.2. Induced Changes onto the NMR Spectrum of the Porphyrin

**Figure 3:**
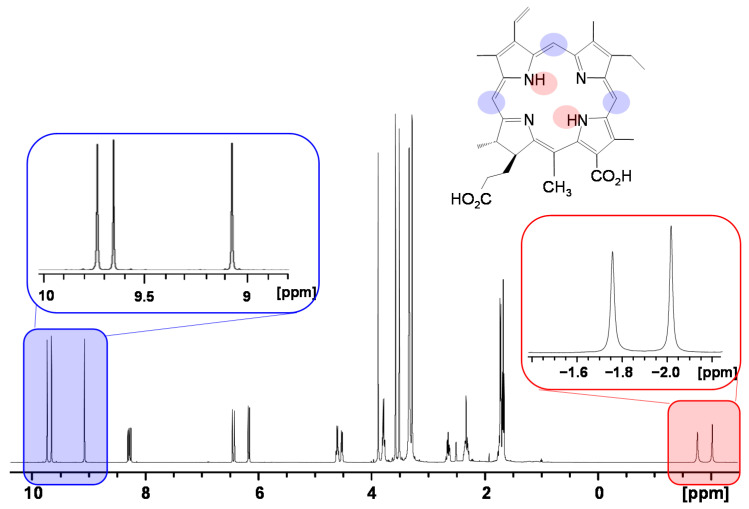
^1^H NMR spectrum of chlorin e4 in DMSO-*d*_6_.

As outlined above, owing to their unique structure porphyrinic compounds give rise to unusual ^1^H NMR spectra in solution [79]. The ring current effect induces a large spread of the ^1^H NMR resonances shifting the inner NH-resonances of free-base porphyrins upfield to values of approximately −2 ppm and the pyrrole- or meso proton resonances located in the periphery of the macrocycle plane downfield to values of approximately 8 ppm and 9–10 ppm, respectively (Figure 3). Since most synthetic polymers as well as bio-macromolecules such as polysaccharides and lipids are devoid of aromatic protons, this spectral region is of specific diagnostic value when studying interactions due to non-overlapping NMR resonances.

In addition to the local magnetic anisotropy that occurs in the frame of a single molecule, the ring current effect also profiles the local magnetic field of the adjacent molecules due to existence of intense intermolecular π–π interactions. Porphyrins exist as monomeric species mostly in unpolar or polar aprotic solvents such as dimethylformamide (DMF) or dimethylsulfoxide (DMSO) and give rise to sharp ^1^H NMR resonances (Figure 3). However, in aqueous solution porphyrin self-aggregation is a well-known phenomenon. The main cause is mostly attributed to π–π interactions between the porphyrin macrocycles and the geometry of the resulting aggregates is determined by the charge distribution in the π system according to the well-known model by Hunter and Sanders [80]. However, substituent effects can also be the dominant forces for porphyrin aggregation. In the early eighties solution structures of porphyrin aggregates have been extensively studied by Abraham, Smith and coworkers applying theoretic models based on ring current-induced NMR aggregation shifts [76,81,82]. Three-dimensional aggregate structures, e.g., formation of *J*- or *H*-aggregates, interplanar distances, orientation, and aggregation maps could be deduced form ^1^H NMR aggregation shift data [83,84]. Thus, depending on the aggregation extent and the magnitude of intermolecular interactions, a single observed nucleus can give rise to resonances at different Larmor frequencies. Therefore, the interpretation of porphyrin NMR spectra and structural elucidation need to be approached with caution. In addition to the large induced shifts, there is a significant effect on the linewidths of the NMR resonances. Both changes in porphyrin ring current-induced shifts (RISs) and line broadening offer measures for interactions with macromolecules.

#### 2.2.3. Induced Changes onto the NMR Spectrum of the Macromolecule

The electron density around the nucleus can be increased or decreased through interactions via chemical bonds or directly through space. The latter type of interaction (through space) allows the detection of nuclei from other molecules in proximity by perturbation of the NMR spectrum of the compound of interest. Monitoring the chemical shifts of the macromolecule (host) upon titration with the small guest molecule provides thus a simple method to probe for intermolecular interactions resolved to molecular segments or even atoms. This chemical shift perturbation (CSP) technique has been widely used for mapping protein-binding sites of ligands [85,86]. The larger the impact of the chemical shift perturbator, the more sensitive is the detection power of the CSP method. The magnitude of perturbation depends on the distance *r* between the interacting species (function of *r*^−3^) and on the physical properties of the small molecule being added. Enhancement of induced changes can be achieved for example by paramagnetic molecules [87] such as lanthanide shift reagents [88] or by molecules with a pronounced magnetic anisotropy as it is encountered in porphyrins [89]. The ring current effect provides a “built-in chemical shift reagent” [79] allowing for a very sensitive detection of interactions with nearby molecules. Based on RISs the spectral perturbation of surrounding macromolecules gives information about sub-molecular sites of interaction and on the relative orientation of the porphyrin macrocycle plane towards its binding partner.

### 2.3. Nuclear Overhauser Enhancement Spectroscopy

The NOE allows detecting through space interactions *d_ij_* between nuclear spins in close proximity and describes the transfer of polarization between nuclear spin populations, mostly ^1^H, via the so-called cross-relaxation phenomenon. In spectra, the NOE manifests itself as the change in the integrated intensity (positive or negative) of one NMR resonance that occurs when another is saturated by irradiation with an RF field. This change in resonance intensity of a nucleus is a consequence of the nucleus being close in space to those directly affected by the RF perturbation [90]. The NOE experiment is therefore a unique method which depends on the spatial proximity between nuclei and measures dipolar couplings as opposed to other NMR techniques which mostly measure scalar couplings (*J*-couplings) among the spins connected through chemical bonds. It can be run as one-dimensional experiment with selective saturation of a single resonance or as two-dimensional experiment (NOE SpectroscopY, NOESY) covering the whole spectral range with the corresponding 2D NOESY pulse sequence [91,92,93]. In small molecules, NOEs may be observed between spins which are up to 4 Å apart [94,95], while the upper limit for large molecules is approximately 5 Å [96]. For large molecules, care must always be taken to avoid the so-called spin-diffusion regime, during which polarization may continue to propagate diffusively among spins until the extra polarization is lost to the lattice (thermal motions) via the spin-lattice relaxation (*T*_1_) process. While being useful for several applications, one disadvantage of spin diffusion is that the size of the observed NOEs is no longer dependent on the spatial proximity between the spins [90,92]. To avoid spin diffusion effects, truncated NOE or transient NOE experiments are performed at short saturation or mixing times enabling to measure NOE build up while minimizing the indirect NOEs [90]. Furthermore, the magnitude and sign of the NOE are determined by the correlation time *τ_c_*, which in turn is a function of the molecular weight (MW). For small molecules the NOE is positive, whereas, for large molecules, it becomes negative. Consequently, at room temperature (298 K) and for intermediate NMR spectrometers, compounds with an intermediate MW of approximately 1000 Da possess a correlation time close to *τ_c_ = 1/ω*_0_ (with *ω*_0_ being the circular resonance frequency), for which the NOE is very weak or can even be zero. In these cases, the rotating frame NOE spectroscopy (ROESY) experiment must be used, as the ROE is always positive; it ranges from maximal 35% (small molecules) to 65% (large molecules) and amounts ~50% when the corresponding NOE is 0 at *τ_c_* = 1/*ω*_0_ [97,98,99].

In summary, the NOE plays an important role in the assignment of NMR resonances and in the determination of the inter- or intra-molecular distances between spins. It is especially useful in the elucidation of structures, steric conformations of organic and biological molecules or host–guest interactions.

### 2.4. NMR Relaxation Times (T_1_ and T_2_)

NMR relaxation phenomena describe the processes by which excited nuclei return to their equilibrium (ground state) distribution. These exponential decay processes can be described and measured using the longitudinal or spin-lattice (*T*_1_) and the transverse or spin–spin (*T*_2_) relaxation times that refer to the recovery of magnetization parallel and decay to zero perpendicular to the direction of the external magnetic field *B*_0_, respectively. The relaxation times of nuclear spins depend on dynamic properties of the corresponding molecules and their interaction with the immediate environment [66]. In longitudinal relaxation (*T*_1_), energy can be transferred to the environment (“lattice”) of the corresponding nucleus by different relaxation mechanisms that can be of paramagnetic, dipolar, or chemical shift anisotropy (CSA) origin [64]. For example, the principle of MRI contrast agents including metalloporphyrins is based on the paramagnetic relaxation enhancement of nearby proton nuclei (shortening of *T*_1_ relaxation time) in biological tissue [36]. *T*_2_ relaxation concerns the loss of transverse magnetization or phase coherence of spins that can be caused through spin–spin interactions and fluctuating magnetic fields. These in turn depend on molecular size and tumbling (Brownian motion), which is characterized by the rotational correlation time *τ_c_*. For macromolecules, molecular motion is slow and *τ_c_* is large, leading to efficient spin–spin relaxation, i.e., short *T*_2_ relaxation times (while the *T*_1_ relaxation time goes through a minimum with increasing *τ_c_*). On the other hand, small fast tumbling molecules with small *τ_c_* have slow relaxation rates (both *T*_1_ and *T*_2_ relaxation times are similar and high) [64].

The relationship between *T*_2_ and *τ_c_* is of particular value for describing the interactions between small and large molecules. Thus, the interaction of porphyrins with biomolecules or the polymeric encapsulation of a porphyrin molecule will lead to a shortening of the spin–spin *T*_2_ relaxation times and can be easily monitored. However, a serious drawback is associated with this interesting property of *T*_2_ relaxation times: Since the observed linewidths of the resonances are directly proportional to the inverse of *T*_2_ (linewidth at half-height ν_1/2_ = 1/π*T*_2_), porphyrins interacting with biomolecules exhibit very broad lines, which reduces sensitivity and can make it very difficult to observe resonances.

### 2.5. Diffusion-Ordered Spectroscopy

The fact that molecular diffusion and diffusion coefficients can be easily studied by NMR methods was realized in the early days of NMR spectroscopy. The easiest and most practical pulse sequence for measuring diffusion coefficients by NMR spectroscopy is the PGSE (Pulsed Gradient Spin Echo) sequence, introduced by Stejskal and Tanner in 1965 [100], actually long before the introduction of 2D NMR spectroscopy. Diffusion NMR measurements have increasingly been used, and the possible applications in solution were summarized in many semantical reviews [101,102,103]. Briefly, the diffusion coefficient, in accordance to the Stokes–Einstein equation (Equation (6)), allows the determination, mostly an approximation, of the hydrodynamic radius, i.e., solvation shell and the size of molecules in solution

(6) $$D=\frac{k_{B}T}{6\pi \eta r}$$

where *k_B_* is the Boltzmann’s constant; *T* the absolute temperature; *η* the dynamic viscosity; and *r* the radius of the spherical particle.

In 1992, it was realized that two-dimensional diffusion spectra can be obtained by incrementing the areas of the gradient pulses in PFG-NMR experiments and transforming the NMR signal amplitudes with respect to the square of this area, resulting in an experiment known as diffusion-ordered NMR spectroscopy (DOSY) [102,104]. DOSY experiments can resolve multiple components based on their different diffusion coefficients giving rise to pseudo-2D spectra correlating the chemical shift (1D spectra) of the components given on the abscissa with the corresponding diffusion coefficients projected on the ordinate. Diffusion coefficients represented on the ordinate are calculated according to the known Stejskal-Tanner equation [100] (Equation (7))

(7) $$\frac{I}{I_{0}}=e^{-D\gamma^{2}g^{2}\delta^{2}\left(\Delta \;-\;\frac{\delta }{3}\right)}$$

where *I*_0_ and *I* are the initial and attenuated NMR signal intensities, *D* the translational diffusion coefficient, γ the gyromagnetic ratio, *g* the gradient strength, *δ* the gradient pulse length and Δ the diffusion time. The DOSY experiment has become a well-established and useful technique for investigating multicomponent mixtures by disentangling the components according to MW and size [105,106,107,108]. Most importantly for the subject of the current review, DOSY can detect intermolecular interactions and the formation of supramolecular systems [101,108,109,110]. This includes homo-association of same molecules as for example in polymeric micelles [103,111] or multi-porphyrin assemblies [112,113] as well as hetero-association of different molecular species. The latter is often applied to monitor the binding of small molecules to larger polymer or protein hosts through alterations in the corresponding diffusion properties. DOSY can thus provide valuable information on complex formation such as the encapsulation of small drugs into polymeric delivery systems [108,114,115]. Here, also, note that the very short *T*_2_ relaxation times of embedded porphyrins and the resulting broad resonances can make it very difficult to record such diffusion experiments.

### 2.6. Heteronuclear NMR Spectroscopy

In addition to the most common observation of ^1^H nuclei in NMR spectroscopy of solutions, there are numerous NMR-active nuclei across the periodic table that can be measured [116,117]. The prerequisite for nuclei to be observable by NMR is that their spin quantum number *I* is unequal to zero, which is the case for all nuclei with an odd number either of both or of the sum of protons and neutrons. Moreover, the suitability of heteronuclear NMR spectroscopy depends on the sensitivity of detection for a given nucleus that is determined by its natural abundance, gyromagnetic ratio and spin quantum number. While nuclei with an asymmetric charge distribution (quadrupolar nuclei, *I* > ½) often give rise to broad NMR resonances, nuclei with half integer spins *I* = ½ are best suited as for example ^1^H, ^13^C, ^15^N, and ^31^P [118]. Since these atoms (H, C, N, and P) belong at the same time to the main constituents in biological material, the corresponding nuclei are most useful in NMR applications of biomolecules such as proteins [119], lipids [120], and carbohydrates [121]. To compensate the low natural abundance of ^13^C (1.1%) or of ^15^N (0.36 %) [116], isotope labeling is often applied, i.e., the enrichment with the corresponding NMR-active nucleus. In addition, the development of NMR hardware (magnets and probeheads) has achieved significant increase in sensitivity for nuclei such as ^13^C or ^15^N [122].

In the study of small molecules interacting with macromolecules, it can be very useful to monitor NMR-active-sensitive nuclei such as ^19^F (*I* = ½) that are not endogenous in biological soft tissue [123], since, compared to ^1^H, the chemical shift range of ^19^F and hetero-nuclei is much larger. For porphyrinic compounds, fluorine substitution [124] or NMR observation of the central metal nuclei in metalloporphyrins [125] thus allows the selective detection of the porphyrin in mixtures with biological or other polymeric compounds without the overload of background signals deriving from the macromolecules.

**Figure 4:**
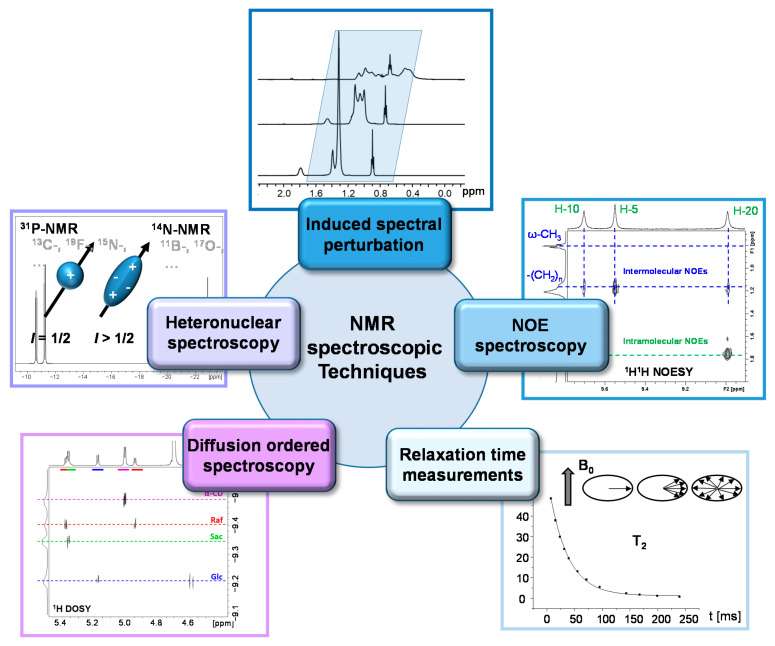
Overview of selected NMR techniques to study porphyrin–macromolecule interactions.

In Figure 4, an overview of the NMR methods explained in Section 2.2, Section 2.3, Section 2.4, Section 2.5 and Section 2.6 is shown. The selected experiments are not exhaustive but represent some of the most frequently applied NMR methods in the study of porphyrinic compounds interacting with macromolecules.

## 3. Applications to Study Porphyrin–Macromolecule Interactions

### 3.1. Biomolecules

#### 3.1.1. Phospholipids (Membrane Models and Liposomal Drug Delivery Vehicles)

Phospholipid membranes belong to the preferential localization sites of porphyrinic PSs in living systems [126,127]. Therefore, interactions with membrane models have been of great interest in the context of PDT. Small unilamellar vesicles (SUVs) composed of phospholipids (PLs) have been used as suitable simplified membrane models providing access to PL-bilayers for solution NMR studies. SUVs are easy to prepare and are small (< 50 nm), fast tumbling systems with some PL mobility within the bilayer that typically give rise to relatively well-resolved proton resonances [128,129]. However, tumbling and in-bilayer mobility are often not fast enough for sufficient averaging of dipolar coupling and direct NMR visibility of small molecules interacting with the PLs. Another contribution originates from strong resonance broadening due to exchange processes between free and PL-bound molecules at intermediate rates on the NMR time scale. The exchange broadening can be reduced by modifications of the conditions (pH, concentration ratios) in solution that alter the exchange kinetics resulting in enhanced NMR visibility of porphyrins associated with PLs in SUVs [130].

**Figure 5:**
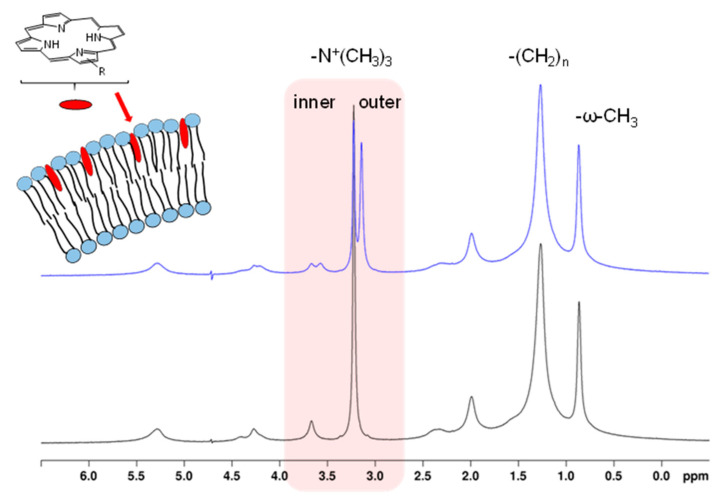
^1^H NMR spectrum (500 MHz) of DOPC SUVs in water before (bottom) and after (top) addition of a Ce6 derivative. The interaction of the chlorin with the DOPC bilayer induces a split of the DOPC choline resonances so that the outer and inner PL layers become distinguishable.

Moreover, indirect changes on the PL resonances such as chemical shift perturbation can provide valuable information on the interactions between porphyrinic molecules and PL-bilayers. Interactions of amphiphilic chlorin e6 (Ce6) derivatives with SUVs consisting of 1,2-dioleoyl-*sn*-glycero-3-phosphocholine (DOPC) resulted in pronounced upfield shifts and splits of the DOPC choline ^1^H resonances due to the porphyrin ring current effect of Ce6 molecules nearby the outer PL bilayer head groups (Figure 5) [131]. This allowed not just the NMR detection of porphyrin membrane association but also of the preferential localization sites along the PL molecules as well as the discrimination between outer and inner PL layers (Figure 5) similar to the impact of lanthanide shift reagents [132]. The magnitude of the *N*-methyl choline resonance split correlated with chlorin concentration up to a level where saturation was reached. The possibility to distinguish between the outer and inner PL monolayers allowed monitoring slow transmembrane kinetics of the Ce6 derivatives by convergence of the split choline resonances over time, which was described as a flip-flop process [131]. The transmembrane kinetics were shown to be strongly pH-dependent for Ce6 derivatives with ionizable carboxylic side chains [133]. Protonation of the carboxylate groups significantly accelerated the transmembrane distribution, whereas Ce6 derivatives bearing carboxylate groups with low p*K*_a_ values were retained in the outer monolayer supported by electrostatic interactions. Accordingly, individual p*K*_a_ values of acidic substituents had a special importance for membrane translocation and could be determined by ^13^C NMR chemical shift titration of the chlorin carboxylate groups [133]. The indirect NMR chemical shift perturbation analysis of 15 different porphyrinic compounds interacting with DOPC vesicles was used to make a classification according to four differently induced NMR patterns of the DOPC -N^+^(CH_3_)_3_- -(CH_2_)_n_-, and -ω-CH_3_-resonances and their time evolutions. From this a relationship between porphyrin structure and type of PL bilayer interaction was proposed where symmetry of substitution, amphiphilicity and overall lipophilicity of the porphyrinic compound were the key factors governing membrane interactions [134]. Another NMR study based on ring current-induced shifts onto PL choline resonances revealed that the porphyrin aggregate structure formed in aqueous solutions has a significant contribution to the initial association with the bilayer surface that is supported by the free accessibility of charged or polar substituents [84].

Unilamellar PL vesicles, also called liposomes, have not just been used as membrane models but also as liposomal delivery vehicles for lipophilic porphyrinic PSs [135,136,137]. Ikeda and coworkers have used ^1^H NMR spectroscopy to monitor the successful encapsulation of a series of tetraphenylporphyrins (TPPs) into the bilayer of DMPC (1,2-dimyristoyl-*sn*-glycero-3-phosphocholine) or egg- phosphocholine (PC) liposomes. They applied an exchange method for improved liposomal drug loading based on a transfer of the porphyrin from a 1:2 CD complex to the liposome. While TPPs forming inclusion complexes with CDs give rise to relatively sharp ^1^H NMR resonances in the aromatic spectral region (see Section 3.2.2), disappearance due to strong resonance broadening upon membrane intercalation (reduced mobility) was used as indicator for successful transfer, which was best for lipophilic TPPs [138,139]. Here, the indirect chemical shift perturbation of the PLs evaluating the relative upfield shifts of the -N^+^(CH_3_)_3_-, -(CH_2_)_n_-, and -ω-CH_3_-resonances was also used to determine the location of the TPPs in the PL bilayer [139].

#### 3.1.2. Proteins

Numerous NMR spectroscopic studies have been applied and reported in the literature for elucidating the structure and function of porphyrin-containing proteins such as hemoglobin, cytochromes and chlorophylls. Early studies of heme proteins have made use of the large hyperfine shifts of paramagnetic metal complexes that are sensitive towards changes in their electronic environment [140]. However, as mentioned in the introduction, this review limits its scope to porphyrinic compounds used as biomedical agents.

For instance, Stojanovic et al. have investigated the interactions between the porphyrin ring and the protein part of porphyrin-containing proteins to better understand their stabilizing role. This study shows that stabilization centers are composed predominantly from nonpolar amino acid residues [141].

Klein-Seetharaman et al. showed using fluorescence and ^1^H and ^19^F NMR spectroscopy that Ce6 weakly binds to rhodopsin with μM affinity. Furthermore, numerous chemical shift changes in the ^1^H-^15^N NMR heteronuclear single quantum coherence (HSQC) spectra of ^15^N-Trp-labeled rhodopsin revealed that Ce6 binding perturbs the entire structure [142].

Two Ce6 derivatives and the barrier function of drug delivery systems towards binding to the serum proteins human serum albumin (HSA) and transferrin (Tf) were monitored by ^1^H NMR spectral appearance of the Ce6 resonances in the aromatic region. Chlorin association with HSA or Tf lead to severe resonance line broadening that was prevented by PVP encapsulation. Block copolymer micelles protected the chlorins from binding to Tf but released them in favor of binding to HSA [143].

#### 3.1.3. Nucleic Acids (DNA, RNA)

Owing to their structures, porphyrins are prone to interact with DNA and RNA, and already in 1979 experimental evidence from binding isotherms, thermal melting profiles, and circular dichroism measurements showed that *meso*-tetrakis (4-*N*-methylpyridyl) porphine (TMPyP) binds to DNA by intercalation [144,145,146]. In 1983, Banville et al. showed that the intercalators TMPyP and Ni(II)TMPyP induced a broad downfield peak in the ^31^P NMR spectrum of DNA and a slight upfield shift of the main peak, while none of these characteristic changes were present in the NMR spectrum of DNA after treatment with the outside-binding porphyrin, Zn(II)TMPyP [147]. Several similar studies involving ^1^H and ^31^P NMR spectroscopy have been summarized by Fiel [148].

More recently, several studies have highlighted how double-stranded DNA or G-quadruplex DNA participates in the reaction with porphyrins, which have contributed to a better understanding of the chemistry of porphyrin models [149].

Another interesting application of NMR is the investigation of the modulation of the PS-quencher unit, which promotes the PDT development, by assessing their interactions with DNA [150,151]. For instance, Hirakawa et al. synthesized a series of water-soluble porphyrin derivatives that target DNA: *meso*-anthryl-tris(*p*-pyridyl)porphyrin (AnTPyP) [152], *meso*-pyrenyl-tris(*N*-methyl-*p*-pyridinio)porphyrin (PyTMPyP) [153], *meso*-(9-anthryl)-tris(*N*-methyl-*p*-pyridinio)porphyrin (AnTMPyP) [154], *meso*-(naphthyl)-tris(*N*-methyl-*p*-pyridinio)porphyrin (NapTMPyP), and TMPyP [155]. In addition to ^1^O_2_, these photosensitization processes could also generate other ROS. Both type I and type II photosensitization processes could occur, depending on the mode that the PS is bound to DNA, i.e., the distance between the PSs and base pairs.

In Table 1, the NMR applications to study porphyrin interactions with biomolecules discussed in Section 3.1.1, Section 3.1.2 and Section 3.1.3 are summarized.

### 3.2. Carrier Polymers

Carrier polymers play an essential role as drug delivery vehicles in the formulation of porphyrinic drugs. NMR spectroscopy is an efficient tool to monitor the conjugation or physical entrapment of small molecular drugs into macromolecular carriers forming NPs. These polymeric delivery systems are mostly NPs with sizes up to 100 nm and their dynamic properties (molecular tumbling and internal motion) are sufficient that they usually give rise to ^1^H NMR spectra in aqueous solution with intense well-resolved resonances.

**Figure 6:**
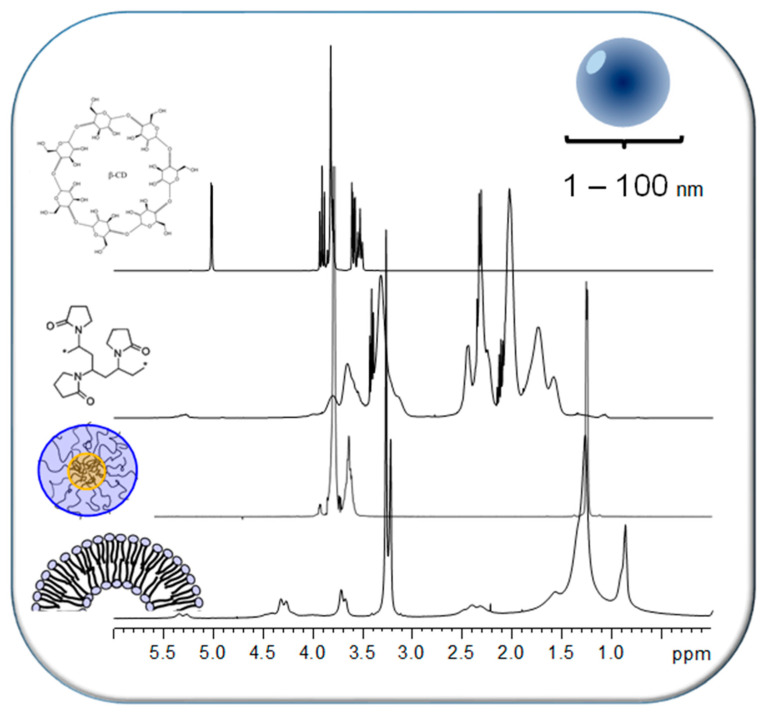
^1^H NMR spectra of selected polymeric nanoparticles used for drug delivery in aqueous solutions, from bottom to top: DOPC SUVs, triblock copolymer (PEG-PPG-PEG) micelles, polyvinylpyrrolidone (PVP) and β-cyclodextrin (CD).

Figure 6 depicts representative examples including phospholipid SUVs, triblock copolymer micelles with polyethylene glycol (PEG) and polypropylene glycol (PPG) blocks, PVP and β-CD. A comprehensive review on the various NMR techniques useful to characterize nanosystems and their interactions with encapsulated drugs as well as with external biologically relevant ligands has been given by Lopez-Cebral et al. [156]. In the subsequent sections, the different polymeric carriers that have been investigated in combination with porphyrinic compounds applying NMR spectroscopic methods are discussed providing a brief overview of studies for each system.

#### 3.2.1. Polyvinylpyrrolidone (PVP)

**Figure 7:**
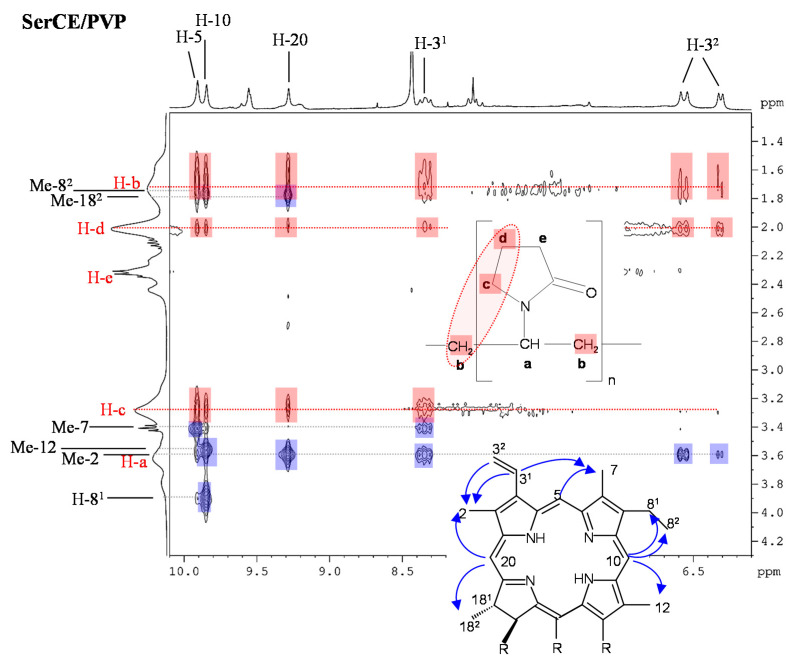
2D ^1^H^1^H NOESY (excerpt) of chlorin e6 serine amide (SerCe) associated with PVP at a SerCe/PVP molar ratio of 3:20 in phosphate buffered saline (PBS). (*Reprinted with permission from: M. Hädener* et al., *J. Phys. Chem. B 2015, 119, 12117−12128.* [163] *Copyright © 2015, American Chemical Society*).

PVP is a widespread polymer used among numerous other applications [157] in the formulation of drugs because of its good water solubility, non-toxicity, inertness and high biocompatibility [158,159]. PVP exists with different MWs and degrees of cross-linking [157] and contains both hydrophilic and hydrophobic functional groups, giving rise to its pronounced versatility with respect to drugs that can be associated with PVP. It is being investigated for the formulation of various porphyrinic PSs. Among those, many studies have focused on the combination of Ce6 with PVP that showed significant enhancement of photodynamic efficiency compared to Ce6 alone and a Ce6–PVP conjugate was approved for PDT under the name Photolone [160]. To determine the origin of PDT enhancement of Ce6–PVP, the interactions between Ce6 and PVP have been analyzed in detail by spectroscopic methods including NMR spectroscopy [161,162,163,164]. In the presence of PVP, pronounced and selective downfield shifts of individual Ce6 ^1^H NMR resonances were observed that indicated disaggregation of the chlorin upon interacting with PVP in aqueous solutions where Ce6 exists as aggregates in the absence of PVP. Chemical shift perturbation of the PVP resonances revealed that Ce6 mainly interacts with the hydrophobic vinyl-backbone of PVP [161,163]. The results could be confirmed by molecular modelling studies based on molecular dynamics of PVP, partial charge distribution in Ce6 and docking analysis of the Ce6–PVP system. In this study, the method for evaluating charge distribution was selected based on linear correlations with experimental ^1^H and ^13^C NMR chemical shifts [162]. PVP was found to have similar disaggregating capability for a series of amino acid derivatives of Ce6 (xCE) bearing either one serine, lysine, tyrosine or arginine residue at the carboxylic acid function of Ce6 as well as for Ce6 mono-6-amino-hexanoic acid amide. All Ce6 derivatives showed aggregation in aqueous solutions to different extents as revealed by their ^1^H NMR spectra, ranging from partly broadened and upfield shifted to strongly broadened or completely disappeared resonances. Interaction with PVP was each indicated by appearance and downfield shifts of the xCE resonances. NMR titration, i.e., monitoring the increasing downfield shifts of the chlorin meso protons as function of PVP concentration, was used to calculate binding constants. Moreover, 2D ^1^H DOSY could prove association of each Ce6 derivative with PVP based on the detection of a common diffusion coefficient for xCE and PVP that was significantly lower than that for the chlorin alone [163]. The observation that xCE and PVP adopt the same dynamic properties with respect to translational motion was also reflected in changes of the transverse relaxation times *T*_2_ and the correlated ^1^H NMR resonance linewidths (inverse proportional to *T*_2_). Another contribution was suggested to derive from motional restriction of the PVP-encapsulated chlorin [163,165]. Regions of preferential intermolecular interactions were derived from 2D ^1^H^1^H NOESY experiments of xCE–PVP mixtures that revealed intermolecular NOE cross peaks between chlorin and PVP resonances. From NOESY-based inter-proton distance calculations it could be concluded that the xCE–PVP complex formation is mainly driven by hydrophobic interactions with participation of the chlorin H-5 and H-10 meso-protons and the PVP H-b and H-c protons (Figure 7) [163].

Similar to the Ce6 amino acid derivatives, the more hydrophobic strongly aggregating chlorin e4 (Ce4) was well encapsulated into PVP, forming stable Ce4–PVP complexes, as was shown by ^1^H NMR chemical shift titration (appearance and increasing upfield shift of Ce4 resonances) and 2D ^1^H DOSY (Ce4 and PVP had a common diffusion coefficient). The PVP-encapsulated Ce6 derivatives Ce4 and serine-amide of Ce6 (SerCe) were also shown to be protected from binding to the external proteins Tf and HSA (see Section 3.1.2) as was indicated by unchanged chlorin ^1^H NMR resonances upon protein addition, whereas severe line broadening occurred in the absence of PVP [143].

In addition to Ce6 derivatives, other porphyrinic compounds as potential PSs have been probed in combination with PVP by NMR spectroscopy. The interaction of the amphiphilic hematoporphyrin derivative (dimegin, DMG) with PVP was studied by induced ^1^H NMR chemical shifts compared to the NMR spectra of the single components. Downfield shifts of the DMG meso-proton resonances indicated disaggregation of the porphyrin that correlated with increased photoactivity. Moreover, it was concluded from PVP chemical shift changes (upfield shifts of all PVP resonances) that not just hydrophobic interactions, that were most pronounced, participated in the binding but also hydrophilic [166,167]. In another study, a series of six different porphyrin derivatives derived either from hematoporphyrin IX (HPIX) or PPIX was monitored with respect to PVP encapsulation by ^1^H NMR spectroscopic analyses of the porphyrin resonances. The results were compared to those obtained from Ce6 derivatives and revealed that PVP has an overall higher capability for disaggregating Ce6 derivatives [165]. This was related to the different aggregate structures and the resulting stability of aggregates that had been deduced for Ce6 and porphyrin derivatives from NMR-derived aggregation maps. The asymmetric substitution pattern of polar and non-polar substituents as well as the nature of the substituents of the Ce6 derivatives lead to aggregate structures that were more easily disrupted than those of the porphyrinic derivatives [84]. Among the investigated porphyrin derivatives, HPIX, deuteroporphyrin IX 2,4-disulfonic acid (DPIXDS), and deuteroporphyrin IX 2,4-disulfonic acid dimethyl ester (DPIXDSME) exhibited relatively good PVP encapsulation based on ^1^H NMR chemical shift titration, ^1^H DOSY and *T*_2_ relaxation time measurements [165].

With the development of theranostic PDT agents, porphyrinic PSs bearing fluorine substituents are very attractive adding the potential for ^19^F-MRI diagnostics. For this purpose, the synthesis of a Zn-phthalocyanine (ZnPc) was reported with 24 fluorine substituents that gave rise to two proximate intense ^19^F NMR signals from the magnetically pseudo-equivalent fluorine atoms of the isomeric mixture. PVP formulation of this hydrophobic F-substituted ZnPc, however, lead to a collapse of the intense ^19^F resonance into a low-intensity very broad signal, which indicated that the ZnPc exists as aggregates in the PVP environment [168].

In Table 2, the NMR applications to study porphyrin interactions with PVP discussed in this section are summarized.

#### 3.2.2. Cyclodextrins (CDs)

**Figure 8:**
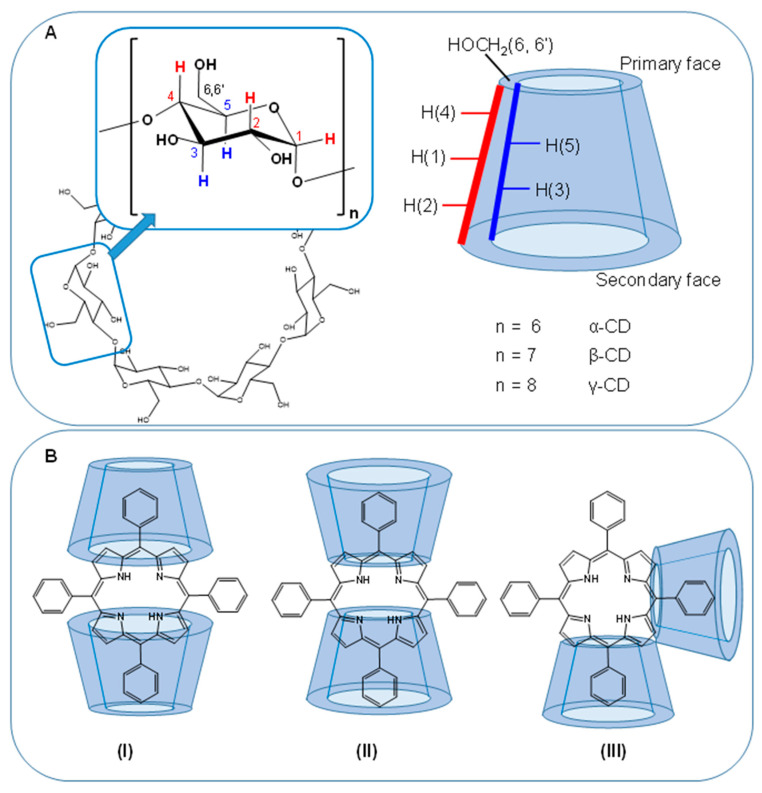
(**A**) Structure of cyclodextrin and the α-1,4-d-glucopyranose unit with atom numbering; sketch of the cone-shaped structure of CDs in which the protons H(3) and H(5) point to the hydrophobic interior of the cavity (blue), the protons H(1), H(2) and (H4) point to the hydrophilic exterior (red), and the protons H(6,6′) are located at the rim of the primary face. (**B**) Examples of possible 2:1 inclusion complexes formed with tetraphenylporphyrins (TPPs): (**I**) inclusion via secondary face, (**II**) inclusion via primary face, opposite (anti) conformation, and (**III**) complex with adjacent (syn) conformation.

CDs are circular oligosaccharides typically consisting of 6 (α-CDs), 7 (β-CDs) or 8 (γ-CDs) α-1,4-D-glucopyranose units. The supramolecular cone-shaped structures form a hydrophobic cavity with the protons H-3 and H-5 on the interior and a hydrophilic surface with H-1, H-2, and H-4 pointing to the outside rendering the CDs water soluble (Figure 8A). The size of the hydrophobic cavity on the primary and secondary face of the cone depends on the number of sugar units and is well suited to form inclusion complexes with small molecules or parts of them (Figure 8B). These features have made CDs very attractive as solubilizers and carriers for drug delivery of a wide range of drugs. Owing to their relatively small size, CDs give rise to well-resolved intense ^1^H NMR spectra (Figure 6, top row). It is therefore not surprising that already three decades ago NMR spectroscopy was extensively applied for characterizing drug-CD inclusion complexes. Schneider et al. have given a comprehensive overview about the various NMR techniques applied to study the structure and binding modes in CD host–guest systems [169].

While the porphyrinic macrocycle is too large for inclusion, peripheral aromatic ring substituents fit well into the hydrophobic cavities of CDs (Figure 8B). Thus, TPPs are predestined to form complexes with CDs and there are numerous studies in the literature that have addressed the interactions between TPP derivatives and CDs by NMR spectroscopic methods. With its atomic resolution, selective changes on the CD nuclei either located in the inner hydrophobic cavity or at the outer hydrophilic surface, NMR spectroscopy is a sensitive method for detecting binding sites of the porphyrinic guest molecules. Depending on the symmetry of the TPP–CD complexes, the TPP pyrrole protons give rise to different numbers of resonance frequencies allowing, e.g., to distinguish oppositely (2 pyrrole signals) or adjacently (4 pyrrole signals) capped TPP (Figure 8B, **II** and **III**). Further, the detection of intermolecular NOEs is an important tool for understanding the spatial arrangement of the porphyrin–CD complexes. Since the MW of porphyrin–CD complexes typically falls into the intermediate regime with MW = 700–1500 Da where the NOE goes through zero, most NMR studies have used the ROESY experiment instead because in ROESY, the NOE is always positive and unequal to zero (see Section 2.3) [169].

Initial studies on phenyl-substituted porphyrin-CD inclusion complexes were inspired by mimicking photosynthetic reaction centers [170] or protein containing porphyrins such as hemoglobin, myoglobin or cytochromes where the CD cavity mimics the protein hydrophobic pockets to obtain water-soluble artificial analogues for mechanistic investigations [171,172,173,174]. Numerous studies have addressed the CD inclusion of *meso*-tetrakis (4-sulfonatophenyl) porphyrin (TPPS_4_) and its metal complexes that exhibit aggregation in aqueous solutions [175,176]. Based on analyses of ^1^H NMR chemical shift changes of the CD- and the TPPS_4_ resonances as well as on intermolecular NOEs it could be concluded that no inclusion complex was formed with α-CD, whereas 2:1 inclusion complexes were formed with β- and γ-CD. In these complexes, the TPPS_4_ sulfonatophenyl substituents were shown to enter the CD cavity through the secondary face in the case of β-CD and through the primary face in the case of γ-CD resulting in different geometries according to the examples shown in Figure 8B (**I**) and (**II**), respectively. In γ–CD complexes, ring-current-induced shifts for H-5 and H-6,6′ were larger compared to β-CD, which in turn had larger shifts of the H-3 protons. Further proof that the TPPS_4_ macrocycle was closer located to the primary face in γ-CD was obtained from ROESY data: In addition to strong NOEs between TPPS_4_ phenyl and inner H-3 and H-5 protons, weak NOEs were also and only detected in γ-CD with H(6,6′) located at the rim of the primary face [176]. Similarly, formation of CD-inclusion complexes could be shown by NMR spectroscopic methods (induced shifts and/or NOE) for the metal complexes Mn(III)TPPS_4_ [177], Zn(II)TPPS_4_ [178,179], and Pd(II)TPPS_4_ [179] with respect to α, β and γ-CD binding affinity and geometry [179]. In these studies, selective changes were also reported for the ^13^C NMR shifts of the inner CD carbon nuclei (C-5 and C-3) of the CD-inclusion complexes [177,179]. Based on weak NOEs between porphyrin protons and protons from the CD exterior surface, non-specific binding of TPPS_4_ monomers or aggregates to the CD outer surface was postulated to coexist with the main inclusion complex.

Compared to the native α-, β- and γ-CDs the corresponding hydroxypropyl (HP) derivatives HP-α-, β- and γ-CDs each yielded similar binding modes but the binding force was stronger in HP-CDs, resulting in more stable complexes with reduced dynamics, i.e., slow dissociation on the NMR time scale [179]. Kano et al. have demonstrated the impact of per-methylation of β-CD using trimethyl-β-CD (TMe-β-CD) that—combined with TPPs bearing anionic substituents in the periphery—formed very stable 2:1 complexes. TPPS_4_ complexation induced much stronger changes in the ^13^C NMR shifts in particular for C-1 and C-4 of TMe-β-CD compared to β-CD. This was related to a higher flexibility of methylated CDs due to the lack of hydrogen-bonds enhancing the binding capability for guest molecules by the “induced fit-type complexation”. ^13^C-*T*_1_ relaxation time measurements provided further insights into the dynamics of the TPPS_4_ (TMe-β-CD)_2_ complex: Slight decrease in the CD- ^13^C-*T*_1_ values indicated reduced motion of the CD scaffold and the rotation of the outer TPPS_4_ phenyl substituents was more inhibited by the two CD-caps than the included ones [180]. Methylated β-CDs (Me-β-CD, HP-β-CD) were also shown by NMR ROESY to form inclusion complexes with TPPS_4_ under acidic conditions where strong J-aggregation dominates. However, for Me-β-CD, an inclusion via the primary face was postulated based on more pronounced changes of the corresponding proton resonances [181]. In the series of *meso*-tetrakis(phenyl)porphyrins with mixed phenyl- and sulfonatophenyl substituents TPPS_3_ and TPPS_2o_ (o = opposite), inclusion complexes with β- and γ-CDs were only formed by insertion of the sulfonatophenyl- but not with the hydrophobic phenyl substituents as could be shown by ROESY experiments. This was explained by intermolecular hydrophobic interactions between phenyl substituents forming lateral porphyrin homo-aggregates that CDs cannot break up as opposed to porphyrin aggregates formed by π–π-stacking. This was evidenced by NMR shift titration and temperature-dependent experiments showing an incomplete disaggregation effect (dissolution of stacked aggregates) for TPPS_3_ and TPPS_2o_ as opposed to TPPS_4_. Type and intensity of intermolecular NOEs were consulted to derive the geometries of the inclusion complexes: In the case of TPPS_3_, both adjacent and opposite sulfonatophenyl substituents were enclosed by CD (exemplified in Figure 8B), and for TPPS_2o_ a slightly tilted CD arrangement was postulated due to coexisting TPP homo-associates via phenyl-ring overlapping. TPPS_2a_ (a = adjacent) did not yield any CD inclusion (absence of NOEs) that was explained by the relatively strong contribution of the two adjacent phenyl substituents to aggregate formation [182]. The presence of adjacently (“syn”) and oppositely (“anti”) capped TPPS_4_-CD inclusion complexes and mixtures thereof could be shown with the application of CD dimers connected through a flexible linker. The different arrangements were deduced from the symmetry/asymmetry of the resulting complexes with adjacent giving rise to four and opposite to two TPPS_4_ -pyrrole proton resonances. In this study, the presence of a 2:2 complex (2 TPPS_4_ and 2 CD dimers with bi-pyridyl-linkers) was also proposed and the structure deduced from NMR-symmetry and dynamic considerations [183]. In the same way, the opposite CD arrangement of a TPPS_4_-TMe-β-CD dimer with a pyridyl-linker serving as hemoglobin model could be confirmed [173].

While most studies focused on CD interactions with TPPS_4_, similar NMR-derived results were obtained for the analogous *meso*-tetrakis(4-carboxyphenyl) porphyrin (TPPC_4_) bearing carboxylate instead of sulfonate substituents [174,179,180]. The importance of charge on the TPP-guest molecule was addressed by several researchers. For TMPyP with four positive charges in the periphery, no inclusion complexes with CDs could be detected [182,184]. However, based on NOEs between TMPyP and the external protons of native and permethylated CDs an external binding mode was suggested. In this study, it was also shown that positive charges on the pyrrole nitrogen atoms of anionic TPPS_4_H_2_^2+^ still enabled the formation of inclusion complexes with CD indicating that only the charge in the periphery determines CD inclusion promoted by electrostatic interactions between anionic substituents and the positively polarized CD cavity [184]. As opposed to TMPyP, the also positively charged corresponding *N*-ethylpyridyl-porphyrin (TEPyP) was clearly shown by NMR-induced chemical shift changes and ROESY data to form inclusion complexes with β-CD and HP-β-CD from the primary face [185]. However, binding affinity to HP-β-CD was much stronger compared to β-CD. Likewise, inclusion of the cationic *p*-phenyl-*O*-(CH_2_)_3_-Py^+^-TPP (TPPOC3Py) into TMe-β-CD was proved by NMR [180]. Moreover, the usage of CD derivatives bearing anionic groups, e.g., sulfonate-CD [184] or sulfobutylether-CD (SBE-CD) [185,186], further promotes binding to cationic TPP derivatives by electrostatic interactions among which NMR data proved the existence of inclusion complexes in the case of SBE-CD.

Hydrophobic interactions were postulated to play a major role in the CD inclusion of TPP derivatives with neutral substituents. In these studies, interactions (NOEs and induced chemical shift changes) were more pronounced with methylated CDs with a more apolar cavity as compared to non-methylated CDs. This could be shown for 5-pyridine-10,15,20-tris-(*p*-chlorophenyl)porphyrin (PyTPP) that hardly interacted with β-CD but formed 1:1 inclusion complexes with TMe-β-CD [187]. A comparative study with di- and tri-methyl CD yielded more intense interactions of 5,10,15,20-tetrakis(4-pyridyl)porphyrin (TPyP) with the trimethylated CD forming a 1:1 complex from the secondary face based on NMR NOE and chemical shift asymmetry indications [188]. Formation of a 1:2 TPyP TMe-β-CD inclusion complex was obtained and confirmed by ^1^H NMR TPP shifts that clearly indicated the symmetry of a bicapped complex with opposite CDs [189]. Similar 1:2 TPP-TMe-β-CD structures were described for neutral TPP derivatives with *p*-hydroxy-phenyl- and *p*-methoxy-phenyl substituents [190] as well as for a TPP bearing an octa-arginine chain on one phenyl ring in which the two opposite non-substituted phenyl rings were included [191].

In the past ten years, most porphyrin-CD related research has converted to the covalent linkage of CD moieties to the porphyrin periphery and NMR spectroscopic methods have been applied for structure characterization and verification. Peripheral CD units can be used to accommodate other molecules combining multiple functions within one complex [192,193,194]. Covalent linkage of several CD units allows to create larger supramolecular structures that—depending on the substitution pattern, symmetry of substitution and nature of substituents—can form larger networks or nanorods suitable for drug delivery [195]. Further, efficiency enhancement could be reached by using CD inclusion complexes as TPP-multiplying unit preventing aggregation at the same time [186,196]. The different binding affinities of TPPS_4_ for β-CD and TMe-β-CD was the basis for the formation of different nano-architectures consisting of alternating TPP units with 4 covalently bound CD units in the periphery bridged byTPPS_4_ units included on opposite sides into free CD cavities. The single tetra-CD-substituted TPP units formed partial, oppositely arranged intramolecular self-inclusion complexes by rotation of the glucose unit directly bound to TPP leaving two free CD cavities as was evidenced by NMR spectroscopic methods. The TPP ^1^H NMR shifts exhibited the pattern of a symmetrical bicapped inclusion complex and NOEs were detected between TPP and CD protons indicating that the CD secondary face was close to the TPP ring. Only in the case of TMe-β-CD the self-inclusion complex was dissolved in favor of TPPS_4_ inclusion (disappearance of intra- and appearance of intermolecular NOEs), providing four binding sites and yielding networks rather than nanorods as in the case of β-CD [197]. Similar self-inclusion complexes of tetra-TMe-CD-TPP derivatives were constructed to obtain water-soluble nanospheres with improved photophysical properties since the nanostructures prevent porphyrin stacking [198].

In Table 3, the NMR applications to study porphyrin interactions with CDs discussed in this section are summarized.

#### 3.2.3. Surfactant Micelles

Micelles are small spheroidal molecular assemblies that spontaneously form from amphiphilic surfactants in aqueous solutions (or in non-polar solutions as reversed micelles) above their characteristic critical micelle concentrations (cmc). Rapid tumbling and high intrinsic mobility in micelles typically give rise to well-resolved, sharp proton resonances. Therefore, NMR spectroscopic techniques are well suited to characterize surfactant micelles and their solutes. For example, the transitions from surfactant monomers to micelles impose changes in the chemical environment and in the dynamic properties that can be monitored by various NMR parameters such as ^1^H- and ^13^C-chemical shifts, *T*_1_- and *T*_2_ relaxation times and diffusion coefficients measured by DOSY experiments [199,200,201].

**Figure 9:**
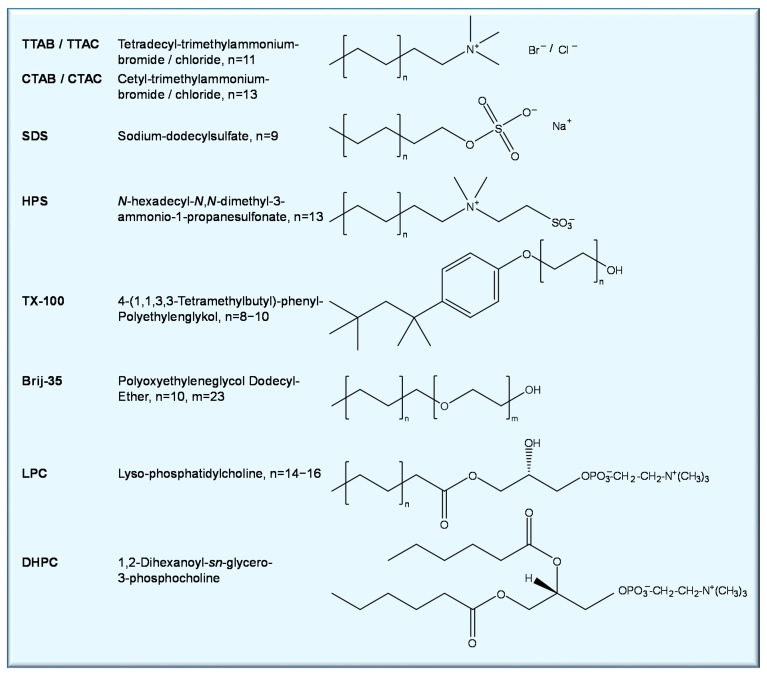
Structures of micelle forming surfactants.

The diverse application of surfactant micelles includes solubilization of hydrophobic molecules and mimicking the anisotropy of biological membranes serving as simplified membrane models [199]. The most frequently applied surfactants have hydrocarbon chains of 12–16 carbon atoms with hydrophilic heads that are either non-ionic, cationic, anionic, or zwitterionic (Figure 9).

In analogy to the TPP–CD complexes (see Section 3.2.2), extensive NMR spectroscopic studies (in the 90ies) have been conducted on heme porphyrins incorporated into CTAB, SDS, or TX-100 (Figure 9) surfactant micelles as models for hemoproteins or enzymes enabling heme studies in a protein-like environment without facing problems of porphyrin aggregation [202,203,204,205,206].

**Figure 10:**
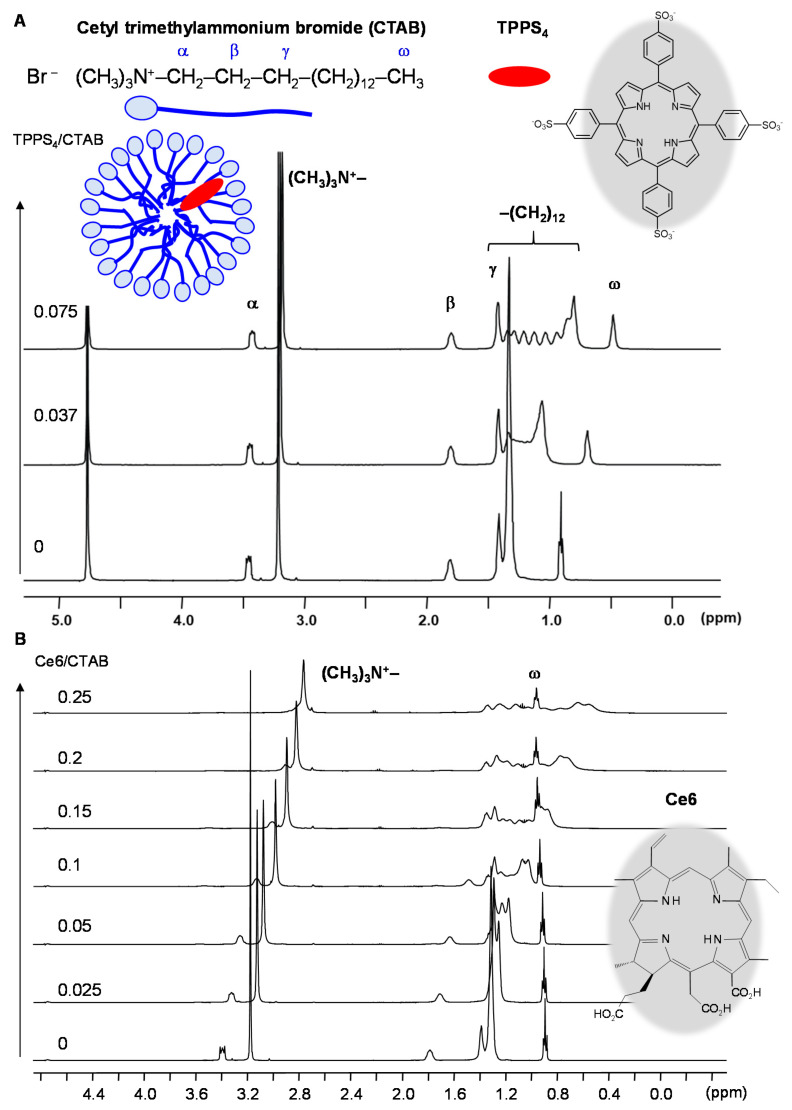
^1^H NMR spectra of CTAB micelles (**A**) in the presence of TPPS_4_ and (**B**) in the presence of chlorin e6 (Ce6) at increasing molar ratios TPPS_4_/Ce6 : CTAB in aqueous buffer solutions (pH = 7.2, CTAB constant concentration 40 mM) (unpublished data).

Utilizing CTAB, SDS and TX-100 micelles as simple membrane models Kadish et al. have shown that the tetra-anionic TPPS_4_ and its metal complexes are solubilized and disaggregated by the cationic CTAB and non-ionic TX-100 micelles but not by anionic SDS micelles demonstrating the importance of electrostatic in addition to hydrophobic interactions. This was evidenced from ^1^H NMR chemical shifts of the TPP and the micellar protons that remained unchanged in the presence of SDS, whereas, for TX-100 and CTAB, ^1^H NMR resonances of TPPS_4_ were downfield shifted and the β-pyrrole resonances coalesced into one single resonance instead of two, which indicated a faster rate of NH-tautomerism of the TPP central NH-groups in the micellar environment. Characteristic ring-current-induced upfield shifts of the TX-100 and CTAB resonances belonging to the hydrophobic core further proved porphyrin insertion [175]. Prominent changes are particularly observed for the CTAB methylene -(CH_2_)_12_ envelope that splits upon TPPS_4_ interaction into single resolved CH_2_ peaks [175,207,208] as is visualized in Figure 10A. This split is also observed in the presence of Ce6. However, as opposed to TPPS_4_, Ce6 exhibits more interactions with the polar head region and does not affect the CTAB terminal methyl-group indicating a micellar insertion of Ce6 that reaches into the hydrophobic region but is more oriented towards the micelle-water interface (Figure 10B, own unpublished data). An analogous NMR study of the cationic TMPyP only yielded insertion into anionic SDS but not into CTAB and TX-100 micelles [209]. Similar NMR results were obtained for a tetra-cationic Pt complex of TMPyP [210]. The lack of interactions with the non-ionic micelles was suggested to be due to the charge delocalization over the entire porphyrin ring in TMPyP reducing the hydrophobic character [209].

The importance of charge and hydrophobic properties for interacting with ionic and non-ionic micelles was also demonstrated on a series of water insoluble TPPs with only one substituted phenyl-ring bearing either a carboxy-, hydroxy-, amino- or nitro group in *para* position. Detailed analysis of micellar ^1^H NMR chemical shifts and the (non-)uniformity, direction and magnitude of induced changes revealed that (i) a certain polarity was required for solubilization in all three micelle types (nitro-phenyl-TPP was not incorporated), (ii) electrostatic repulsive forces inhibited micelle insertion, (iii) peripheral hydroxy-groups promoted micelle insertion, since hydroxy-phenyl-TPP reached highest intra-micellar concentration and was more oriented towards the micelle water interface compared to the other TPP derivatives [211].

The interaction of TPPS_4_ as free base or metal complex with cationic and zwitterionic micelles was the subject of further comprehensive NMR spectroscopic studies. By varying the surfactant concentration below and above the cmc it could be shown that TPPS_4_ forms premicellar aggregates with CTAB, whereas micellized monomers exist above cmc [212]. Similar premicellar aggregates were also reported for cationic TMPyP and pyridiniumpropoxy-TPP in the presence of SDS below cmc [210,213].

Based on ^1^H NMR chemical shift, *T*_1_ relaxation time and NOE data, it was shown that TPPS_4_ localizes in the hydrophobic core of cationic CTAC and zwitterionic HPS micelles, whereas, in LPC micelles (for the surfactant structures see Figure 9), the polar head region is also involved in TPP interactions. The split methylene resonance (as exemplified in Figure 10A) allowed the determination of NOE buildup rates for TPP protons through dipolar interactions with CTAC protons in different chain positions in addition to the choline and terminal methyl-group in truncated and transient NOE experiments. Taking intramolecular differences in surfactant mobility (rotational correlation times) into account, the different NOE rates could provide an interaction profile along the surfactant molecules [207]. Metal complexes of TPPS_4_ (Fe(III), Zn(II)) exhibited similar localization sites in CTAC, HPS and in the non-ionic TX-100 and Brij-35 (Figure 9) micelles evidenced by largest upfield shifts for the terminal surfactant protons [208,214]. More recent studies have reported the insertion of diaxial Sn(IV)– and Co(III)–TPPS_4_ complexes into the hydrophobic region of CTAB micelles as concluded from selective 1D NOE experiments [215,216].

To study the interactions with a series of Ce6, HPIX and PPIX derivatives, short-chain PL micelles obtained from DHPC (Figure 9) were used as mobile PL-membrane models with enhanced dynamics, making it possible to observe encapsulated porphyrin resonances by ^1^H NMR spectroscopy in the non-overlapping aromatic region. Based on the appearance of the ^1^H NMR chemical shifts of the porphyrinic compounds it could be concluded that DHPC-micelles have a high capability to include and break up even strongly aggregating Ce6 derivatives, whereas this was not the case for the porphyrin compounds. This indicated that not the extent but the structure of aggregates as well as the accessibility of peripheral polar substituents play key roles in porphyrin membrane insertion [84].

In Table 4, the NMR applications to study porphyrin interactions with surfactant micelles discussed in this section are summarized.

#### 3.2.4. Block Copolymer Micelles (BCMs)

**Figure 11:**
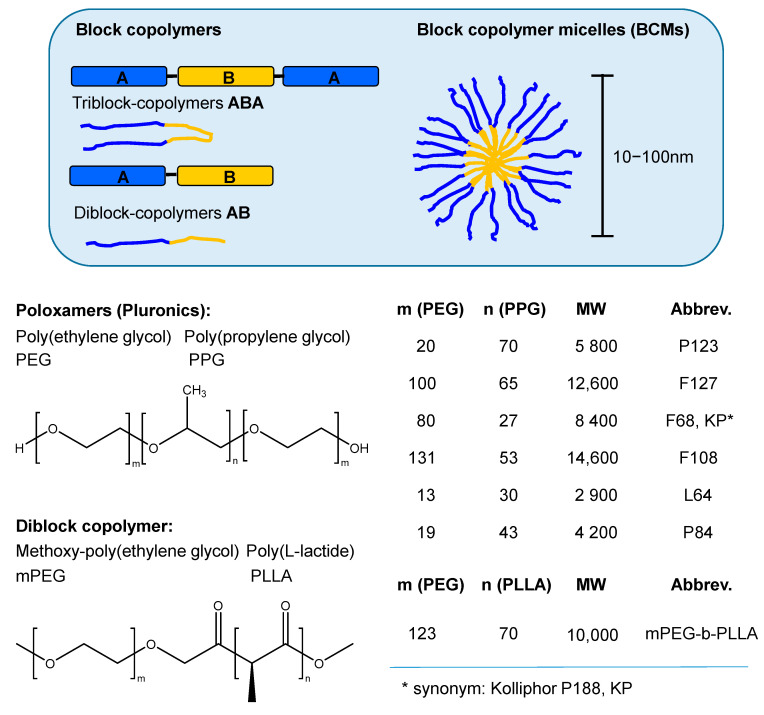
Structures of block copolymers forming micelles discussed in the current review.

BCMs have gained much interest as versatile delivery systems for a wide range of drugs including porphyrinic PSs [54,55] and several recent comprehensive review articles have been devoted to the chemistry, properties and applications of BCMs [54,217,218,219,220]. The BCM forming units are biocompatible, mostly synthetic polymers consisting of a hydrophilic block forming the micellar shell and a hydrophobic block forming the micellar core (Figure 11). The blocks can be arranged in two (A-B-type diblock copolymers) or three alternating segments (A-B-A-type triblock copolymers) and their chain lengths, ratio and overall MW determine the size and morphology of BCMs. Owing to their dynamic properties as relatively small fast tumbling systems with a high degree of internal motional freedom of the flexible polymer chains [221], BCMs are very well applicable for NMR spectroscopic characterizations giving rise to intense ^1^H NMR resonances in aqueous solution (Figure 6). The micelle formation process from block copolymer unimers induces changes not just in the diffusion properties but also in the ^1^H- and ^13^C-chemical shifts, resonance linewidths and relaxation rates of the polymeric nuclei rendering NMR a suitable method to monitor micellization [222,223,224,225,226]. Several studies either probing newly designed or commercial block copolymers for PS delivery have exploited NMR spectroscopy to verify polymer structures and to determine block sizes and MW of the polymers by quantitative NMR peak integration [227]. However, since the current review focuses on *interaction* studies, the corresponding work will not be included here. Since physical entrapment is frequently the mechanism of drug loading, polymer–drug interactions are of utmost importance for the loading capability, stability of the system and drug release. For this, NMR techniques including 1D and 2D NOE spectroscopy, NMR relaxation time and diffusion (DOSY) measurements are particular powerful tools in the description of the intermolecular interactions.

PEG is the most often encountered hydrophilic component of BCMs and in particular the triblock copolymers PEG-PPG-PEG, the so-called poloxamers or Pluronics (BASF trade name) [228], have been extensively explored as carriers for porphyrinic PSs [54,55,161,162,166,229,230,231]. BCM encapsulation is a strategy to enhance porphyrin delivery towards targeted tissue or profile the pharmacokinetics and pharmacodynamics of bioactive molecules. PEG grafting reduces mechanisms of biological recognition that lead to fast clearance via opsonization by the reticuloendothelial system (RES) [232,233]. In addition to its utilization in micellar form as carrier for low-MW drugs, PEGylation is also used as covalent modifier of biological macromolecules [232,234,235].

Steinbeck et al. have conducted extensive NMR studies on the interaction of TPPS_4_ with Pluronic P123 (PEG_20_PPG_70_PEG_20_, Figure 11) micelles at different temperatures. They analyzed ^1^H NMR chemical shift changes of TPPS_4_-P123 mixtures to determine the localization of the porphyrin along the polymer chains. Since the PPG methine and methylene resonances partially overlap with the PEG methylene peak they applied ^1^H^13^C HSQC for obtaining a better resolution. At low temperatures (<20 °C) TPPS_4_ exhibited strong interactions with the PPG-blocks, whereas, at higher temperatures (>35 °C), stronger interactions with the PEG part were observed. Sites of interactions were indicated by selective upfield shifts as well as—in part—inhomogeneous broadening either of the PEG- or PPG-polymer resonances and simultaneous downfield shifts of the TPPS_4_ resonances. The interactions were confirmed by corresponding intermolecular rotating frame NOEs (ROEs). The temperature dependence of interactions was explained by a shift towards a dehydrated, more hydrophobic nature of the polymer chains with increasing temperature. Detailed diffusion data including HSQC-resolved diffusion measurements at different temperatures and diffusion times lead to the conclusion that at <20 °C small TPPS_4_-P123 aggregates were formed, which at higher temperatures were in fast exchange with TPPS_4_ present in the larger micelles [236].

In analogy to TPPS_4_, the corresponding tetra-sulfonated Pc, ZnPcS_4_, as well as the perfluorinated ZnPcF_16_ were reported to preferentially localize in the PEG corona region of mPEG-*b*-PLLA (Figure 11) diblock copolymer micelles as was derived from induced ^1^H NMR chemical shift changes of the PEG moiety and NOE data (in the case of ZnPcS_4_). On the contrary, the non-substituted congener ZnPc lacked interactions with PEG and was localized in the hydrophobic micellar core. The mPEG-*b*-PLLA micelles were suggested to form a relatively rigid core region since the PLLA and the encapsulated ZnPc proton resonances were strongly broadened and only became NMR-visible after dissolution of the assembly by acetone addition [237].

The interaction of the hematoporphyrin derivative DMG [166] and Ce6 [161] with F127 (Figure 11) Pluronic micelles or solely with PEG as homo-polymer was each probed by ^1^H NMR spectroscopy. Both porphyrinic compounds exhibited disaggregation in the presence of the BCMs or PEG alone as was indicated by downfield shifts of the porphyrin/chlorin meso-proton, propionic acid and vinyl side chain protons, respectively. Both compounds were suggested to localize preferentially in the hydrophobic core regions of the BCMs due to induced upfield shifts and partial broadening specifically of the F127-PPG resonances. However, disaggregation capability and observed upfield shifts of the PEG resonance in the case of mixing with the sole homo-polymer also proved a significant affinity of both DMG and Ce6 for the more hydrophilic PEG blocks so that a micellar location close to the PEG-PPG interface was suggested [161,166].

In a comparative NMR study, BCMs formed by the poloxamer Kolliphor P188 (KP, Figure 11) also exhibited good disaggregation capability for Ce6 and four of its amino acid derivatives SerCe, lysine-, tyrosine- and arginine-amide of Ce6 (LysCe, TyrCe, and ArgCe) but a divergent one for porphyrinic compounds. Unlike HPIX, DPIXDS and its methylester DPIXDSME, DPIX, PPIX and iso-HPIX (iHPIX) were not disaggregated. The results were similar to those obtained for PVP and were attributed to the more stabilized aggregate structures of the porphyrinic compared to the Ce6 compounds. Chemical shift titration of the Ce6 derivatives resulted in progressive downfield shifts of selected (meso- and vinyl-) Ce6 ^1^H-resonances with increasing KP concentration yielding uptake curves that levelled off at molar ratios of 3:10 (Ce6/KP). DOSY measurements of mixtures with KP revealed a dynamic exchange of Ce6 derivatives between the bulk phase and the micellar environment, since the Ce6 resonances had *D* values representing a weighted average between free and BCM-associated chlorin molecules. With increasing KP concentration this equilibrium was shifted towards micellized Ce6 compounds as the corresponding *D* values gradually decreased and levelled off. In this way, DOSY titration could be used to determine the BCM loading process and to derive uptake curves similar to the chemical shift titration experiments. The enhanced exchange dynamics were assumed to be responsible for the fact that only very weak intermolecular NOEs between the Ce6 derivative SerCe and the PPG methyl protons were observable. However, a strong and SerCe-concentration-dependent selective perturbation of the PPG methyl resonance (upfield shift and line broadening) clearly indicated the micellar core region as main interaction site [165].

**Figure 12:**
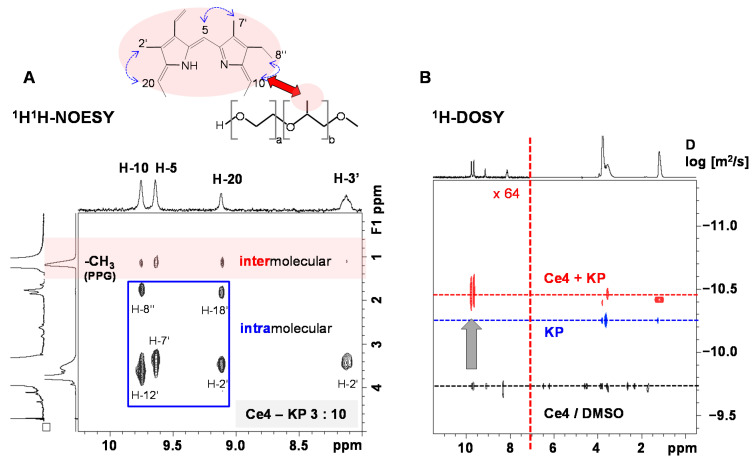
(**A**) ^1^H ^1^H-NOESY spectrum of Ce4–KP in phosphate buffered saline (PBS, molar ratio 3:10); *intra*molecular NOE cross peaks are visible for the Ce4 meso proton resonances (marked by the blue square) and *inter*molecular NOEs are visible between the Ce4 meso resonances and the methyl resonance of KP (highlighted in red and indicated in the structures); (**B**) overlay of ^1^H-DOSY spectra of 3 mM Ce4 in DMSO (shown in black), 10 mM KP in PBS (shown in blue), and Ce4–KP in PBS at a molar ratio of 3:10 (shown in red). For Ce4–KP (3:10) the DOSY spectrum and the projection spectrum are scaled up by a factor of 64 in the region between 7 and 11 ppm. T = 310 K. *(Reprinted from Gjuroski* et al. *Journal of Controlled Release, 316, (2019), 150-167 [143], © 2019 Published by Elsevier B.V., with permission from Elsevier)*.

Similar NMR studies with Ce4 proved that the micellar host–guest system (KP micelles as host and Ce4 as guest) could be stabilized by increased hydrophobicity and reduced water solubility of the guest molecule significantly repelling the dynamic exchange process [143]. In this system, more intense NOEs could be observed (Figure 12A). The three NOE cross peaks shown in Figure 12A, which arose from the interaction of the PPG methyl group of KP with the meso-H from Ce4, indicated localization in the micellar hydrophobic core with the hydrophobic part of the porphyrin molecule contributing to a stabilization effect of the Ce4–KP micellar assembly in aqueous medium. Moreover, the detection of negative NOEs (at a magnetic field strength of 11.74 T) demonstrated that the chlorin molecules behaved like large, slowly tumbling systems, proving their micellar association [90]. In addition, DOSY experiments not just confirmed the encapsulation of Ce4 in KP micelles but also indicated a lack of dynamic exchange (Figure 12B). The same diffusion coefficient (red line) was measured for the meso-protons from Ce4 (9.5 ppm) and for the -CH_2_- and CH_3_-groups (3.7 and 1.2 ppm, respectively) from KP showing that the Ce4–KP complex diffused as a single particle. Moreover, the corresponding *d* value of Ce4–KP (red line) was smaller than the *d* value of pure KP (blue line) as well as Ce4 (black line) alone, pointing out an increased hydrodynamic radius (according to Stokes–Einstein equation, Equation (6)) of Ce4-loaded micelles [143].

Detailed NMR studies with five Ce6 derivatives of increasing hydrophobicity from SerCe, Ce4, Ce6 monoethylene diamine monoamide (CeMED), Ce6 dimethylester (CeDM) towards Ce6 trimethylester (CeTM) demonstrated the limitation of the poloxamer BCM encapsulation capability with respect to very hydrophobic strongly aggregating Ce6 compounds. Five different BCM forming Pluronic polymers with increasing HLB (hydrophilic-lipophilic balance) values and different block ratios and MWs were probed applying KP, F108, F127, L64 and P84 (Figure 11). Quantitative chlorin encapsulation was assessed by appearance and integration of the chlorin ^1^H resonances in the aromatic spectral region. Loading efficiency inversely correlated with chlorin hydrophobicity for each type of polymer. However, there was no correlation with the HLB value of the polymers, whereas the number of PPG blocks independent of the PEG part played a role as well as within certain limits the size of the PEG corona. Again, upfield shifts, broadening, asymmetric shapes, and the extent of chemical shift perturbation of the polymer PPG nuclei were very sensitive towards chlorin interaction and only in L64 and P84 BCMs also the PEG resonance was shifted indicating contributions from the micellar corona region. Dynamic properties of the chlorin BCM systems were addressed by DOSY, *T*_1_ and *T*_2_ relaxation time measurements. According to the DOSY spectra, a part of the systems exhibited dynamic exchange as indicated by fraction-weighted average *d* values for the chlorin nuclei in mixtures, whereas, for another part, the chlorin was more tightly bound to the micellar core with the chlorin molecules adopting the same diffusion properties as their micellar hosts [226].

Chlorin *T*_1_ and *T*_2_ relaxation times exhibited an overall reduction upon interaction with any of the BCM types due to an increase in their molecular correlation time *τ_c_* [226]. Binding to the host molecule (the BCMs) increases the relaxation rates of the guest (the Ce6 derivatives) due to imposed tumbling motion from the host, i.e., longer correlation time resulting in shorter *T*_2_ [238]. However, not only the tumbling motion, also the spins in the vicinity and the distance between the spins in interactions influence the spin relaxation dynamics [239]. While BCM encapsulation was accompanied by chlorin disaggregation, the differential *T*_2_ values of the chlorin protons were equalized as they entered the homogeneous micellar environment. In chlorin aggregates protons of overlapping regions had strongly reduced relaxation times compared to those not experiencing the ring current of neighboring chlorin macrocycles. Similarly, for the pure poloxamer micelles, *T*_2_ values of the PPG spins were an order of magnitude shorter than those of the PEG units reflecting the spatial crowding in the micellar core region, whereas, in the outer corona, motional freedom is more and spin–spin interactions are less pronounced.

Chlorin encapsulation induced slight increases in the polymer *T*_1_ and *T*_2_ relaxation times that was explained by a disturbance of the molecular order. Further, it was postulated that the internal BCM flexibility as reflected in relaxation rates conduced to loading efficiency but not to stability (stronger binding) [226].

This was also supported by a comparison of PVP and KP micelles used as carriers: Although KP micelles had a smaller diffusion coefficient, i.e., larger hydrodynamic radius compared to PVP, implying longer molecular correlation times (*τ_c_*) and shorter *T*_2_, chlorin association to the PVP matrix lead to stronger drop in chlorin *T*_2_ values as compared to KP. This was ascribed to the stiffness of the PVP matrix for which despite a smaller size than KP-BCMs dipole–dipole interactions were more pronounced. In accordance with this, chlorin-binding constants were an order of magnitude higher for PVP than for KP [165].

In Table 5, the NMR applications to study porphyrin interactions with block copolymer micelles discussed in this section are summarized.

## 4. Conclusions

The interest in porphyrins as medical drugs is still growing and polymeric nanoplatforms have become an inevitable part of efficient delivery to the target tissue. With the development of theranostic agents, the systems have become complex and their characterization at the atomic level more demanding. While the optical properties of porphyrin-based drugs as phototoxic agents, i.e., their light absorbance and energy conversion, will remain the major focus in the analytical assessment, interactions with macromolecules are of equal high importance. Such interactions can significantly modify the photophysical properties and also determine the thermodynamic and kinetic stability of porphyrin formulations.

With this review, our aim was to provide an overview of studies that have applied NMR spectroscopy to address the interactions of biomedical-relevant porphyrinic compounds with macromolecules in solution. We have shown that NMR spectroscopy can offer a wealth of complementary information, thanks to its versatility, its accessibility to a vast range of dynamic properties, and its possibility to measure interactions with atomic resolution. For instance, the strong ring current inherent to porphyrins makes this class of compounds particularly suited for easy NMR detection of induced changes upon interaction with their large host molecules. In addition, NMR spectroscopy is non-destructive, highly reproducible and can be directly applied even to complex mixtures without the need of sample preparation steps except for the dissolution in an adequate solvent. Nevertheless, NMR spectroscopy also has several limitations: A low sensitivity compared to mass spectrometry or fluorescence spectroscopy and severe resonance line broadening can make certain large molecules or components with restricted, anisotropic mobility non-observable by NMR spectroscopy of solutions. Here, solid-state NMR techniques using magic angle spinning (MAS) and/or high resolution MAS (HR-MAS) NMR of semi-solids are better suited, but these two techniques have not been treated in this review in order to limit its scope and length. Yet, we hope that readers can realize through the examples given in this review, such as porphyrins interacting with CDs and surfactant micelles, that most nanoparticles can be studied by high resolution NMR in solution. It is a matter of fact, confirmed by a survey of the recent literature, that NMR spectroscopy is still rather underrepresented despite its enormous potential. This review may promote and encourage future studies involving porphyrins or any other type of small-molecule drugs interacting with macromolecules to make use of the advantages of NMR spectroscopy as powerful and versatile analytical tool.

## Figures and Tables

**Table 1:** Summary of NMR interaction studies between porphyrins and biomolecules.

Porphyrin (Guest)	Macromolecule (Host)	NMR Technique	Result	Ref
*Phospholipids*				
Ce6 Ce6 derivatives	DOPC-SUVs	^1^H NMR chem. shift perturbation of host Time-dependent ^1^H NMR chem. shift perturbation of host	Ce6 attached to PL-bilayer head group Transmembrane kinetics of Ce6 (flip-flop) pH dependence of kinetics	[131,133]
Ce6, Ce6 derivatives PPIX, DPIX, HPIX and derivatives	DOPC-SUVs	^1^H NMR chem. shift perturbation of host	Porphyrin aggregate structure determines membrane interaction	[84]
Ce6 derivatives PPIX, DPIX, HPIX and derivatives TPP derivatives	DOPC-SUVs	^1^H NMR chem. shift perturbation of host Time-dependent ^1^H NMR chem. shift perturbation of host	Different patterns of bilayer localization and transmembrane kinetics depending on porphyrin structure and substitution Patterns used for classification of membrane interactions	[134]
TPP Zn-TPP	DMPC liposomes	^1^H NMR spectral appearance of guest	Transfer from CD complex to liposome	[138]
TPP	Egg-PC liposomes	^1^H NMR chem. shift perturbation of host	Liposomal localization (hydrophobic core)	[139]
*Proteins*				
Ce6	Bovine rhodopsin ^19^F-/^15^N-Trp-labeled rhodopsin	^1^H-, ^19^F- and ^15^N-NMR chem. shift perturbation of host ^1^H-^15^N NMR HSQC ^1^H NMR spectral appearance of guest	Weak binding of Ce6 to rhodopsin localized at cytoplasmic domain	[142]
Ce6 SerCe	HSA Tf	^1^H NMR spectral appearance of guest	Binding to both HSA and Tf PVP encapsulation prevents binding BCM encapsulation prevents only Tf binding	[143]
*Nucleic acids*				
TMPyP, Ni(II)TMPyP, Zn(II)TMPyP	DNA	^31^P NMR chem. shift perturbation of host	TMPyP, Ni(II)TMPyP intercalate, Zn(II)TMPyP binds to the outside of DNA	[147]
Cationic TMPyP derivatives	DNA	^31^P-, ^1^H NMR chem. shift perturbation of host	Review: Three binding modes (intercalation, outside binding, outside binding with self-stacking)	[148]

**Table 2:** Summary of NMR interaction studies between porphyrins and PVP.

Porphyrin (Guest)	Macromolecule (Host)	NMR Technique	Result	Ref
Ce6	PVP (MW 25 kDa)	^1^H NMR chem. shift perturbation of host ^1^H NMR spectral appearance of guest	Ce6 mainly interacts with the hydrophobic vinyl-backbone of PVP Disaggregation upon interaction with PVP	[161]
Ce6	PVP (MW 40 kDa)	^1^H NMR spectral appearance of guest	Disaggregation upon interaction with PVP	[164]
Ce6 SerCe LysCe TyrCe ArgCe Ce6-amino-hexanoic amide	PVP (MW 10 kDa)	^1^H NMR spectral appearance of guest ^1^H NMR chem. shift titration with host ^1^H DOSY of host–guest mixture 2D ^1^H^1^H NOESY of host–guest mixture	Disaggregation upon interaction with PVP Determination of binding constant Host and guest have same diffusion properties Identification of host and guest protons in close proximity	[163]
SerCe	PVP (MW 10 kDa)	*T_2_* relaxation time measurements of host and guest	Change and assimilation of dynamic properties of host and guest Motional restriction of guest	[163,165]
Ce4	PVP (MW 10 kDa)	^1^H NMR spectral appearance of guest ^1^H NMR chem. shift titration with host ^1^H DOSY of host–guest mixture	Disaggregation upon interaction with PVP Determination of binding constant Host and guest have same diffusion properties	[143]
Ce4, SerCe	PVP (MW 10 kDa), HSA, Tf	^1^H NMR spectral appearance of guest	PVP-encapsulated guest is protected from protein binding	[143]
DMG	PVP (MW 40 kDa)	^1^H NMR spectral appearance of guest ^1^H NMR chem. shift perturbation of host	Disaggregation upon interaction with PVP Hydrophobic and hydrophilic interactions between host and guest	[166,167]
PPIX, DPIX, HPIX and derivatives	PVP (MW 10 kDa)	^1^H NMR spectral appearance of guest	Different extent of disaggregation upon PVP interaction	[165]
HPIX, DPIXDS, DPIXDSME	PVP (MW 10 kDa)	^1^H NMR chem. shift titration with host ^1^H DOSY of host–guest mixture *T_2_* relaxation time measurements of host and guest	Determination of binding curves Host and guest have same diffusion properties Restricted mobility of encapsulated guest	[165]
Fluorinated ZnPc (ZnPcF_24_)	PVP	^19^F NMR spectral appearance of guest	Guest exists as aggregate in PVP	[168]

**Table 3:** Summary of NMR interaction studies between porphyrins and cyclodextrins.

Porphyrin (Guest)	Macromolecule (Host)	NMR Technique	Result	Ref
TPPS_4_	α-, β-, γ-CD	^1^H NMR chem. shift perturbation of host 2D ^1^H^1^H ROESY of host–guest mixture	2:1 (CD:TPPS_4_) inclusion complexes with β- and γ-CD, no complex with α-CD β-CD: through secondary face γ -CD: through primary face	[176]
TPPS_4_, Mn(III)TPPS_4_	α-, β-, γ-CD	^1^H and ^13^C NMR chem. shift perturbation of host 2D ^1^H^1^H ROESY of host–guest mixture	Strongest binding for β-CD	[177]
Zn(II)TPPS_4_	β-CD	^1^H NMR chem. shift perturbation of host	Formation of inclusion complex	[178]
Zn(II)TPPS_4_ Pd(II)TPPS_4_ TPPC_4_	β-, γ-CD HP-β-CD HP-γ-CD	^1^H and ^13^C NMR chem. shift perturbation of host 2D ^1^H^1^H ROESY of host–guest mixture	Formation of inclusion complexes: (HP)β-CD: through secondary face (HP)γ -CD: through primary face Weak binding to CD exterior	[179]
TPPS_4_ TPPOC3PS^a^ TPPC_4_ TPPOC3Py	β-Cd TMe-β-CD	^1^H and ^13^C NMR chem. shift perturbation of host 2D ^1^H^1^H ROESY ^1^H NMR spectral appearance of guest ^13^C-*T_1_* relaxation time of host and guest	β-CD, TMe-β-CD: anionic porphyrin guests binding more favorable than cationic Disaggregation; formation of trans-type 2:1 complexes with TMe-β-CD TPPS_4_: stronger binding to TMe-β-CD than to β-CD Motional restriction of CD and TPP-phenyl rings (less pronounced inside cavity)	[180]
TPPS_4_ Acidic conditions	β-CD Me-β-CD HP-β-CD	^1^H and ^13^C NMR chem. shift perturbation of host 2D ^1^H^1^H ROESY ^1^H NMR spectral appearance of guest	β-CD, HP-β-CD: inclusion via secondary face Me-β-CD: inclusion via primary face Disaggregation of J-aggregates upon complexation	[181]
TPPS_4_ TPPS_3_ TPPS_2o_ TPPS_2a_ TMPyP	β-, γ-CD	^1^H NMR spectral appearance of guest ^1^H NMR chem. shift perturbation of host 2D ^1^H^1^H ROESY	Inclusion complexes formed with all but TPPS_2a_ and TMPyP TPPS_4_, TPPS_3_, TPPS_2o_: Partial disaggregation Sulfonatophenyl- but not phenyl-group included in the case of mixed substituents Inclusion via secondary face for β-CD and primary for γ-CD	[182]
TPPS_4_ TPPC_4_	CD dimers with flexible spacers	^1^H NMR spectral appearance of guest ^1^H NMR chem. shift perturbation of host	Adjacently (“syn”) and oppositely (“anti”) capped TPPS_4_	[183]
TMPyP TPPS_4_H_2_^2+^	α-, β-, γ-Cd TMe-β-CD SO_3_-β-CD DiMeSO_3_-β-CD	^1^H NMR chem. shift perturbation of host ^1^H NMR spectral appearance of guest ^1^H NMR chem. shift titration with host 2D ^1^H^1^H ROESY	TMPyP: External binding to native CDs and TMe-β-CD TPPS_4_H_2_^2+^: Inclusion with β-CD via secondary and with γ-CD via primary face Fast exchange between free/complex form	[184]
TEPyP	β-CD HP-β-CD SBE-CD	^1^H NMR chem. shift perturbation of host ^1^H NMR spectral appearance of guest 2D ^1^H^1^H ROESY	Inclusion with β-CD and HP-β-CD from the primary face	[185]
TMPyP	SBE-CD	^1^H NMR spectral appearance of guest	Complex formation with SBE-CD Fast exchange between free/complex form	[186]
PyTPP	β-CD Tme-β-CD	^1^H NMR chem. shift perturbation of host ^1^H NMR spectral appearance of guest	Inclusion with TMe-β-CD (from the primary face) but hardly with β-CD	[187]
TPyP	HP-β-CD Di-Me-β-Cd TMe-β-CD	^1^H NMR spectral appearance of guest 2D ^1^H^1^H NOESY	1:1 complex with TMe-β-CD from the secondary face	[188]
TPyP	TMe-β-CD	^1^H NMR spectral appearance of guest at different pH	1:2 (TPyP:CD) complex pH-dependent release (acidic condition)	[189]
TPP, TPPC_4_ THPP^b^, TAPP^c^ TMeOPP^d^	TMe-β-CD	^1^H NMR spectral appearance of host–guest mixture	All form 1:2 (TPP:CD) inclusion complexes with TMe-β-CD	[190]
octa-arginine-TPP (R8-TPP)	TMe-β-CD	^1^H NMR chem. shift titration with host ^1^H NMR spectral appearance of guest 2D ^1^H^1^H ROESY	Trans-type 1:2 (TPP:CD) inclusion complex with the non-substituted phenyl groups via secondary face Slow free/complex exchange rate	[191]
TPPS_4_	ZnTPP- DAPM-β-CD^e^	^1^H NMR spectral appearance of host–guest mixture 2D ^1^H^1^H NOESY ^1^H-DOSY	ZnTPP- β-CD forms self-inclusion and inclusion complexes with TPPS_4_ yielding vesicles and networks	[195]
Mn(III)TPP PEGylated	bridged bis(TMe-β-CD)	2D ^1^H^1^H NOESY	Inclusion complexes, formation of supramolecular polymers	[196]
TPPS_4_	ZnTPP-, DAPM-β-CD^e^ DAPM-TMe-β-CD^e^	^1^H NMR spectral appearance of host–guest mixture 2D ^1^H^1^H NOESY	ZnTPP- β-CD and -TMe-β-CD form self-inclusion and inclusion complexes; the latter are dissolved in favor of TPPS_4_ inclusion Formation of networks and nanorods	[197]

^a^ TPPOC3PS: *p*-phenyl-*O*-(CH_2_)_3_-*p*-phenyl-(*S*O_3_)^−^-tetra-phenylporphyrin; ^b^ THPP: meso-tetrakis(4-hydroxyphenyl) porphyrin; ^c^ TAPP: meso-tetrakis(4-aminophenyl) porphyrin; ^d^ TMeOPP: meso-tetrakis(4-methoxyphenyl) porphyrin; ^e^ DAPM: 6-deoxy-6-azidopermethyl.

**Table 4:** Summary of NMR interaction studies between porphyrins and surfactant micelles.

Porphyrin (Guest)	Macromolecule (Host)	NMR Technique	Result	Ref
TPPS_4_ Zn(II)TPPS_4_ Cu(II)TPPS_4_ VO^2+^TPPS_4_	CTAB SDS TX-100	^1^H NMR chem. shift perturbation of host ^1^H NMR spectral appearance of guest	Encapsulation in hydrophobic core of CTAB and TX-100 micelles Disaggregation in CTAB, TX-100 SDS promotes aggregation, no insertion	[175]
TPPS_4_	CTAC HPS LPC	^1^H NMR chem. shift perturbation of host ^1^H NMR spectral appearance of guest 1D NOE spectroscopy ^1^H *T_1_* relaxation times	CTAC and HPS micelles: TPPS_4_ localizes in the hydrophobic core Reduced molecular mobility of TPPS_4_ LPC micelles: TPPS_4_ intercalates involving the polar head region Fast exchange between free and bound states	[207]
TPPS_4_ Fe(III)TPPS_4_ Zn(II)TPPS_4_	CTAC LPC	^1^H NMR chem. shift perturbation of host; pH dependence ^1^H NMR spectral appearance of guest ^1^H *T_1_* relaxation time	TPPS_4_, Zn(II)-, Fe(III)TPPS_4_: Incorporate into CTAC and LPC micelles in the core region pH-dependent aggregation of Fe(III)TPPS_4_ in micelles	[208]
TMPyP Zn(II)TMPyP Cu(II)TMPyP VO^2+^TMPyP	CTAB SDS TX-100	^1^H NMR chem. shift perturbation of host ^1^H NMR spectral appearance of guest	All solubilized by SDS micelles but not by CTAB and TX-100 Monomerization in SDS	[209]
Pt(Cy_2_dim)Me]_4_(TpyP) ^a^	SDS TX-100	^1^H NMR chem. shift perturbation of host	Location in hydrophobic region of SDS micelles; Low solubility in TX-100	[210]
*p*-(OH)-phenyl-TPP *p*-(COOH)-phenyl-TPP *p*-(NH_2_)-phenyl-TPP *p*-(NO_2_)-phenyl-TPP	TTAB SDS TX-100	^1^H NMR chem. shift perturbation of host ^1^H NMR chem. shift titration with guest ^1^H NMR spectral appearance of guest	Insertion inhibited by electrostatic repulsion *p*-(NO_2_)-TPP not inserted in any micelles *p*-(OH)-phenyl-TPP inserted in all micelles most efficiently	[211]
TPPS_4_	CTAB	^1^H NMR spectral appearance of guest ^1^H NMR chem. shift titration with host	Below cmc: Premicellar aggregates Above cmc: Micellar insertion and monomerization	[212]
TPPOC3Py	SDS	^1^H NMR chem. shift perturbation of host	Below cmc: Premicellar aggregates Above cmc: Intercalation among SDS chains, monomerization	[213]
Fe(III)TPPS_4_ Zn(II)TPPS_4_,	CTAC HPS Brij-35 TX-100	^1^H NMR chem. shift perturbation of host ^1^H NMR spectral appearance of guest	Fe(III)TPPS_4_, Zn(II)TPPS_4_, embedded in hydrophobic core of the micelles	[214]
Co(III)TPPS_4_(imidazole)_2_	CTAB	1D selective NOE spectroscopy	Closer location near the CTAB micellar core	[215]
Sn(IV)TPPS_4_(OH)_2_ Sn(IV)TPPS_4_(Met)_2_ Sn(IV)TPPS_4_(Tyr)_2_	CTAB	1D selective NOE spectroscopy	Closer location near the CTAB micellar core	[216]
Ce6, Ce4 Ce6 derivatives DPIX, PPIX, HPIX and derivatives	DHPC	^1^H NMR spectral appearance of guest	Chlorin derivatives: Monomerized in DHPC micelles Porphyrin derivatives: Only weak interactions, no disaggregation	[84]

^a^ Cy_2_dim: dicyclohexyldiimine.

**Table 5:** Summary of NMR interaction studies between porphyrins and block copolymer micelles.

Porphyrin (Guest)	Macromolecule (Host)	NMR Technique	Result	Ref
TPPS_4_	P123	^1^H NMR chem. shift perturbation of host ^1^H^13^C HSQC ^1^H NMR spectral appearance of guest 1D ROE spectroscopy DOSY (HSQC-res.) *T*-dependent NMR	<20 °C: TPPS_4_ strong interactions with PPG units Small TPPS_4_-P123 aggregates >35 °C: stronger interactions with PEG units TPPS_4_-P123 micelles in fast exchange with smaller TPPS_4_-P123 aggregates	[236]
ZnPc ZnPcS_4_ ZnPcF_16_	mPEG-b-PLLA	^1^H NMR chem. shift perturbation of host ^1^H NMR spectral appearance of guest 1D NOE spectroscopy	ZnPc localized in micellar core ZnPcS_4_ and ZnPcF_16_ localize in micellar corona	[237]
DMG	F127 pure PEG	^1^H NMR chem. shift perturbation of host ^1^H NMR spectral appearance of guest	Disaggregation DMG localizes in hydrophobic core of F127 micelles DMG forms complex with PEG	[166]
Ce6	F127 pure PEG	^1^H NMR chem. shift perturbation of host ^1^H NMR spectral appearance of guest	Disaggregation Ce6 localizes in the PEG-PPG-interface region of F127 micelles	[161]
Ce6 SerCe, LysCe, TyrCe, ArgCe DPIX DPIXDS, DPIXDSME PPIX HPIX, iHPIX	KP	^1^H NMR spectral appearance of guest ^1^H NMR chem. shift perturbation of host ^1^H NMR chem. shift titration with host ^1^H DOSY 1D NOE spectroscopy	Chlorins: disaggregation Porphyrins: only HPIX, DPIXDS, DPIXDSME disaggregate Determination of binding curves Dynamic exchange between free and micellar state Preferential localization in micellar core	[165]
Ce4	KP	^1^H NMR spectral appearance of guest ^1^H NMR chem. shift perturbation of host ^1^H NMR chem. shift titration with host ^1^H DOSY 2D NOESY	Disaggregation Localization in hydrophobic core of KP micelles Lack of dynamic exchange	[143]
SerCe Ce4 CeMED CeDM CeTM	KP F108 F127 L64 P84	^1^H NMR spectral appearance of guest ^1^H NMR chem. shift perturbation of host ^1^H DOSY ^1^H *T_1_* / *T_2_* relaxation	Loading efficiency inversely correlated with chlorin hydrophobicity Interaction mainly with hydrophobic core Differently pronounced dynamic exchange Disaggregation accompanied by changes in the dynamics	[226]

## Data Availability

Data sharing is not applicable to this article.

## Appendix A. The Following Abbreviations were used in the Text

AnTMPyP, meso-(9-anthryl)-tris(*N*-methyl-*p*-pyridinio)porphyrin; AnTPyP, meso-anthryl-tris(*p*-pyridyl)porphyrin; ArgCe, arginine-amide of Ce6; BCM, block copolymer micelle; Brij-35, polyoxyethylene glycol dodecyl-ether; CD, cyclodextrin; Ce4, chlorin e4; Ce6, chlorin e6; CeDM, chlorin e6 dimethylester; CeMED, chlorin e6 monoethylene diamine monoamide; CeTM, chlorin e6 trimethylester; cmc, critical micelle concentration; CSA, chemical shift anisotropy; CSP, chemical shift perturbation; CTAB, cetyl-trimethylammonium-bromide; CTAC, cetyl-trimethylammonium-chloride; Cy_2_dim, dicyclohexyldiimine; DAPM, 6-deoxy-6-azidopermethyl; DHPC, 1,2-Dihexanoyl-*sn*-glycero-3-phosphocholine; DMF, dimethylformamide; DMG, dimegin; DMPC, 1,2-dimyristoyl-*sn*-glycero-3-phosphocholine; DMSO, dimethylsulfoxide; DOPC, 1,2-dioleoyl-*sn*-glycero-3-phosphocholine; DOSY, diffusion-ordered spectroscopy; DPIXDS, deuteroporphyrin IX 2,4-disulfonic acid; DPIXDSME, deuteroporphyrin IX 2,4-disulfonic acid dimethyl ester; DSS, 2,2-dimethyl-2-silapentane-5-sulfonate; HLB, hydrophilic-lipophilic balance; HPIX, hematoporphyrin IX; HPS, *N*-hexadecyl-*N*,*N*-dimethyl-3-ammonio-1-propanesulfonate; HP-β-CD, hydroxypropyl-β-cyclodextrin; HR-MAS, high resolution magic angle spinning; HSA, human serum albumin; HSQC, heteronuclear single quantum coherence; iHPIX, iso- hematoporphyrin IX; KP, Kolliphor P188; LPC, lyso-phosphatidylcholine; LysCe, lysine-amide of chlorin e6; MAS, magic angle spinning; Me-β-CD, methylated β-cyclodextrin; MOF, metal–organic frameworks; MRI, magnetic resonance imaging; MW, molecular weight; NapTMPyP, meso-(naphthyl)-tris(*N*-methyl-*p*-pyridinio)porphyrin; NIR, near infrared; NMR, nuclear magnetic resonance; NOE, nuclear Overhauser effect; NOESY, nuclear Overhauser effect spectroscopy; NP, nanoparticle; PAI, photoacoustic imaging; PBS, phosphate buffered saline; Pc, phthalocyanine; PCI, photochemical internalization; PC, phosphocholine; PDD, photodynamic diagnosis; PDT, photodynamic therapy; PEG, polyethylene glycol; PET, positron emission tomography; PFG, pulsed field gradient; PGSE, pulsed gradient spin echo; PL, phospholipid; PPG, polypropylene glycol; PPIX, protoporphyrin IX; PS, photosensitizer; PVP, polyvinylpyrrolidone; PyTMPyP, meso-pyrenyl-tris(*N*-methyl-*p*-pyridinio)porphyrin; PyTPP, 5-pyridine-10,15,20-tris-(*p*-chlorophenyl)porphyrin; RES, reticuloendothelial system; RF, radio frequency; RIS, ring current-induced shift; ROE, rotating frame nuclear Overhauser effect; ROESY, rotating frame nuclear Overhauser effect spectroscopy; ROS, reactive oxygen species; R8-TPP, octa-arginine-TPP; SBE-CD, sulfobutylether-cyclodextrin; SDS, sodium-dodecylsulfate; SerCe, serine-amide of chlorin e6; SUV, small unilamellar vesicle; TAPP, meso-tetrakis(4-aminophenyl) porphyrin; TEPyP, *N*-ethylpyridyl-porphyrin; Tf, transferrin; THPP, meso-tetrakis(4-hydroxyphenyl) porphyrin; TMe-β-CD, trimethyl-β-cyclodextrin; TMeOPP, meso-tetrakis(4-methoxyphenyl) porphyrin; TMPyP, meso-tetrakis (4-*N*-methylpyridyl) porphine; TMS, tetramethylsilane; TPP, tetraphenylporphyrin; TPPC_4_, meso-tetrakis (4-carboxyphenyl) porphyrin; TPPOC3PS, *p*-phenyl-*O*-(CH_2_)_3_-*p*-phenyl-(SO_3_)^−^-tetra-phenylporphyrin; TPPOC3Py, *p*-phenyl-*O*-(CH_2_)_3_-Py^+^-tetra-phenylporphyrin; TPPS_4_, meso-tetrakis (4-sulfonatophenyl) porphyrin; TPyP, 5,10,15,20-tetrakis(4-pyridyl)porphyrin; TX-100, Triton X-100, 4-(1,1,3,3-tetramethylbutyl)-phenyl-polyethylene glycol; TyrCe, tyrosine-amide of chlorin e6; xCE, amino acid derivatives of chlorin e6; ZnPc, zinc-phthalocyanine; ZnPcF_16_, perfluorinated zinc-phthalocyanine; ZnPcS_4_, tetra-sulfonated zinc-phthalocyanine.

## References

[1] Lemberg R., Ƶechmeister L. (1954). Porphyrins in Nature. Fortschritte der Chemie Organischer Naturstoffe/Progress in the Chemistry of Organic Natural Products/Progrés dans la Chimie des Substances Organiques Naturelles.

[2] Williams R.J.P. (1956). The Properties Of Metalloporphyrins. Chem. Rev..

[3] Boucher L.J., Melson G.A. (1979). Coordination Chemistry of Porphyrins. Coordination Chemistry of Macrocyclic Compounds.

[4] Battersby A.R. (2000). Tetrapyrroles: The pigments of life. Nat. Prod. Rep..

[5] Lesage S., Xu H.A.O., Durham L. (1993). The occurrence and roles of porphyrins in the environment: Possible implications for bioremediation. Hydrol. Sci. J..

[6] Bryant D.A., Hunter C.N., Warren M.J. (2020). Biosynthesis of the modified tetrapyrroles-the pigments of life. J. Biol. Chem..

[7] Poulos T.L. (2014). Heme enzyme structure and function. Chem. Rev..

[8] Latunde-Dada G.O., Caballero B., Finglas P.M., Toldrá F. (2016). Iron: Biosynthesis and Significance of Heme. Encyclopedia of Food and Health.

[9] Scheer H., Grimm B., Porra R.J., Rüdiger W., Scheer H. (2006). An Overview of Chlorophylls and Bacteriochlorophylls: Biochemistry, Biophysics, Functions and Applications. Chlorophylls and Bacteriochlorophylls: Biochemistry, Biophysics, Functions and Applications.

[10] Tanaka A., Tanaka R., Grimm B. (2019). Chapter Six—The biochemistry, physiology, and evolution of the chlorophyll cycle. Advances in Botanical Research.

[11] Hunter C.N., Thurnauer F.D.M.C., Beatty J.T. (2009). The Purple Phototrophic Bacteria.

[12] Graham R.M., Deery E., Warren M.J. (2009). Vitamin B12: Biosynthesis of the Corrin Ring. Tetrapyrroles: Birth, Life and Death.

[13] Kadish K.M., Smith K.M., Guilard R., Kadish K.M., Smith K.M., Guilard R. (2003). The Porphyrin Handbook. The Porphyrin Handbook.

[14] Min Park J., Lee J.H., Jang W.-D. (2020). Applications of porphyrins in emerging energy conversion technologies. Coord. Chem. Rev..

[15] Costa E., Silva R., Oliveira da Silva L., de Andrade Bartolomeu A., Brocksom T.J., de Oliveira K.T. (2020). Recent applications of porphyrins as photocatalysts in organic synthesis: Batch and continuous flow approaches. Beilstein J. Org. Chem..

[16] Martínez-Díaz M.V., de la Torre G., Torres T. (2010). Lighting porphyrins and phthalocyanines for molecular photovoltaics. Chem. Commun..

[17] Huang H., Song W., Rieffel J., Lovell J.F. (2015). Emerging applications of porphyrins in photomedicine. Front. Phys..

[18] Imran M., Ramzan M., Qureshi A.K., Khan M.A., Tariq M. (2018). Emerging Applications of Porphyrins and Metalloporphyrins in Biomedicine and Diagnostic Magnetic Resonance Imaging. Biosensors.

[19] Tsolekile N., Nelana S., Oluwafemi O.S. (2019). Porphyrin as Diagnostic and Therapeutic Agent. Molecules.

[20] Castano A.P., Demidova T.N., Hamblin M.R. (2004). Mechanisms in photodynamic therapy: Part one-photosensitizers, photochemistry and cellular localization. Photodiagnosis Photodyn. Ther..

[21] Castano A.P., Demidova T.N., Hamblin M.R. (2005). Mechanisms in photodynamic therapy: Part three-Photosensitizer pharmacokinetics, biodistribution, tumor localization and modes of tumor destruction. Photodiagnosis Photodyn. Ther..

[22] Lin Y., Zhou T., Bai R., Xie Y. (2020). Chemical approaches for the enhancement of porphyrin skeleton-based photodynamic therapy. J. Enzym. Inhib. Med. Chem..

[23] Costa L.D., Costa J.I.T., Tomé A.C. (2016). Porphyrin Macrocycle Modification: Pyrrole Ring-Contracted or -Expanded Porphyrinoids. Molecules.

[24] Lindsey J.S., Hsu H.C., Schreiman I.C. (1986). Synthesis of tetraphenylporphyrins under very mild conditions. Tetrahedron Lett..

[25] Lo P.-C., Rodríguez-Morgade M.S., Pandey R.K., Ng D.K.P., Torres T., Dumoulin F. (2020). The unique features and promises of phthalocyanines as advanced photosensitisers for photodynamic therapy of cancer. Chem. Soc. Rev..

[26] Mody T.D., Sessler J.L. (2001). Texaphyrins: A new approach to drug development. J. Porphyr. Phthalocyanines.

[27] Teo R.D., Hwang J.Y., Termini J., Gross Z., Gray H.B. (2017). Fighting Cancer with Corroles. Chem. Rev..

[28] Dougherty T.J. (2002). An update on photodynamic therapy applications. J. Clin. Laser Med. Surg..

[29] Kwiatkowski S., Knap B., Przystupski D., Saczko J., Kędzierska E., Knap-Czop K., Kotlińska J., Michel O., Kotowski K., Kulbacka J. (2018). Photodynamic therapy—Mechanisms, photosensitizers and combinations. Biomed. Pharmacother..

[30] Cieplik F., Deng D., Crielaard W., Buchalla W., Hellwig E., Al-Ahmad A., Maisch T. (2018). Antimicrobial photodynamic therapy—What we know and what we don’t. Crit. Rev. Microbiol..

[31] Carvalho C.M.B., Tomé J.P.C., Faustino M.A.F., Neves M.G.P.M.S., Tomé A.C., Cavaleiro J.A.S., Costa L., Alves E., Oliveira A., Cunha Â. (2009). Antimicrobial photodynamic activity of porphyrin derivatives: Potential application on medical and water disinfection. J. Porphyr. Phthalocyanines.

[32] Jocham D., Stepp H., Waidelich R. (2008). Photodynamic Diagnosis in Urology: State-of-the-Art. Eur. Urol..

[33] Zumbraegel A., Bichler K.-H., Krause F.S., Feil G., Nelde H.J., Atala A., Slade D. (2003). The Photodynamic Diagnosis (PDD) for Early Detection of Carcinoma and Dysplasia of the Bladder. Bladder Disease, Part A: Research Concepts and Clinical Applications.

[34] Fritsch C., Lang K., Neuse W., Ruzicka T., Lehmann P. (1998). Photodynamic diagnosis and therapy in dermatology. Skin Pharmacol. Appl. Skin Physiol..

[35] Cheng H.L., Haedicke I.E., Cheng W., Tchouala Nofiele J., Zhang X.A. (2014). Gadolinium-free T1 contrast agents for MRI: Tunable pharmacokinetics of a new class of manganese porphyrins. J. Magn. Reson. Imaging.

[36] Ni Y. (2008). Metalloporphyrins and Functional Analogues as MRI Contrast Agents. Curr. Med. Imaging Rev..

[37] Nasim V., Amir R.J. (2015). An Overview of Labeled Porphyrin Molecules in Medical Imaging. Recent Pat. Top. Imaging.

[38] Yap S.Y., Price T.W., Savoie H., Boyle R.W., Stasiuk G.J. (2018). Selective radiolabelling with 68Ga under mild conditions: A route towards a porphyrin PET/PDT theranostic agent. Chem. Commun..

[39] Merkes J.M., Zhu L., Bahukhandi S.B., Rueping M., Kiessling F., Banala S. (2020). Photoacoustic Imaging Probes Based on Tetrapyrroles and Related Compounds. Int. J. Mol. Sci..

[40] Abuteen A., Zanganeh S., Akhigbe J., Samankumara L.P., Aguirre A., Biswal N., Braune M., Vollertsen A., Röder B., Brückner C. (2013). The evaluation of NIR-absorbing porphyrin derivatives as contrast agents in photoacoustic imaging. Phys. Chem. Chem. Phys..

[41] Zhang Y., Lovell J.F. (2012). Porphyrins as theranostic agents from prehistoric to modern times. Theranostics.

[42] Jenni S., Sour A. (2019). Molecular Theranostic Agents for Photodynamic Therapy (PDT) and Magnetic Resonance Imaging (MRI). Inorganics.

[43] Jerjes W., Theodossiou T.A., Hirschberg H., Høgset A., Weyergang A., Selbo P.K., Hamdoon Z., Hopper C., Berg K. (2020). Photochemical Internalization for Intracellular Drug Delivery. From Basic Mechanisms to Clinical Research. J. Clin. Med..

[44] Norum O.-J., Selbo P.K., Weyergang A., Giercksky K.-E., Berg K. (2009). Photochemical internalization (PCI) in cancer therapy: From bench towards bedside medicine. J. Photochem. Photobiol. B: Biol..

[45] Chen J., Zhu Y., Kaskel S. (2020). Porphyrin-Based Metal-Organic Frameworks for Biomedical Applications. Angew. Chem. Int. Ed. Engl..

[46] Kim J., Jo Y.-U., Na K. (2020). Photodynamic therapy with smart nanomedicine. Arch. Pharmacal Res..

[47] Moret F., Reddi E. (2017). Strategies for optimizing the delivery to tumors of macrocyclic photosensitizers used in photodynamic therapy (PDT). J. Porphyr. Phthalocyanines.

[48] Sztandera K., Gorzkiewicz M., Klajnert-Maculewicz B. (2020). Nanocarriers in photodynamic therapy-in vitro and in vivo studies. WIREs Nanomed. Nanobiotechnol..

[49] Calixto G.M., Bernegossi J., de Freitas L.M., Fontana C.R., Chorilli M. (2016). Nanotechnology-Based Drug Delivery Systems for Photodynamic Therapy of Cancer: A Review. Molecules.

[50] Montaseri H., Kruger C.A., Abrahamse H. (2020). Recent Advances in Porphyrin-Based Inorganic Nanoparticles for Cancer Treatment. Int. J. Mol. Sci..

[51] Düzgüneş N., Piskorz J., Skupin-Mrugalska P., Goslinski T., Mielcarek J., Konopka K. (2018). Photodynamic therapy of cancer with liposomal photosensitizers. Ther. Deliv..

[52] Derycke A.S.L., de Witte P.A.M. (2004). Liposomes for photodynamic therapy. Adv. Drug Del. Rev..

[53] Huang H.C., Mallidi S., Obaid G., Sears B., Tangutoori S., Hasan T., Hamblin M.R., Avci P. (2015). 23—Advancing photodynamic therapy with biochemically tuned liposomal nanotechnologies. Applications of Nanoscience in Photomedicine.

[54] Demazeau M., Gibot L., Mingotaud A.-F., Vicendo P., Roux C., Lonetti B. (2020). Rational design of block copolymer self-assemblies in photodynamic therapy. Beilstein J. Nanotechnol..

[55] Nascimento B.F.O., Pereira N.A.M., Valente A.J.M., Pinho E., Melo T.M.V.D., Pineiro M. (2019). A Review on (Hydro)Porphyrin-Loaded Polymer Micelles: Interesting and Valuable Platforms for Enhanced Cancer Nanotheranostics. Pharmaceutics.

[56] Pehlivan E.G., Ek Y., Topkaya D., Tazebay U.H., Dumoulin F. (2019). Effect of PVP formulation on the in vitro photodynamic efficiency of a photosensitizing phthalocyanine. J. Porphyr. Phthalocyanines.

[57] Chin W.W.L., Heng P.W.S., Thong P.S.P., Bhuvaneswari R., Hirt W., Kuenzel S., Soo K.C., Olivo M. (2008). Improved formulation of photosensitizer chlorin e6 polyvinylpyrrolidone for fluorescence diagnostic imaging and photodynamic therapy of human cancer. Eur. J. Pharm. Biopharm..

[58] Mazzaglia A., Micali N., Scolaro L.M., Sciortino M.T., Sortino S., Villari V. (2010). Design of photosensitizer/cyclodextrin nanoassemblies: Spectroscopy, intracellular delivery and photodamage. J. Porphyr. Phthalocyanines.

[59] Ben Mihoub A., Larue L., Moussaron A., Youssef Z., Colombeau L., Baros F., Frochot C., Vanderesse R., Acherar S. (2018). Use of Cyclodextrins in Anticancer Photodynamic Therapy Treatment. Molecules.

[60] Gouterman M., Dolphin D. (1978). 1—Optical Spectra and Electronic Structure of Porphyrins and Related Rings. The Porphyrins.

[61] Ernst R.R., Bodenhausen G., Wokaun A. (1987). Principles of Nuclear Magnetic Resonance in One and Two Dimensions.

[62] Becker E.D., Bradley R.B. (1959). Effects of ‘‘Ring Currents’’ on the NMR Spectra of Porphyrins. J. Chem. Phys..

[63] Scheer H., Katz J.J., Smith K.M. (1975). Nuclear magnetic resonance spectroscopy of porphyrins and metalloporphyrins. Porphyrins and Metalloporphyrins.

[64] Keeler J. (2010). Understanding NMR Spectroscopy.

[65] Günther H. (2013). NMR Spectroscopy: Basic Principles, Concepts, and Applications in Chemistry.

[66] Friebolin H. (2010). Basic One- and Two-Dimensional NMR Spectroscopy. 5th edition ed..

[67] Karplus M. (1959). Contact Electron-Spin Coupling of Nuclear Magnetic Moments. J. Chem. Phys..

[68] Karplus M. (1963). Vicinal Proton Coupling in Nuclear Magnetic Resonance. J. Am. Chem. Soc..

[69] Minch M.J. (1994). Orientational dependence of vicinal proton-proton NMR coupling constants: The Karplus relationship. Concepts Magn. Reson..

[70] Pretsch E., Clerc T., Seibl J., Simon W. (1981). Protonenresonanzspektroskopie. Tabellen zur Strukturaufklärung Organischer Verbindungen Mit Spektroskopischen Methoden.

[71] Pauling L. (1936). The Diamagnetic Anisotropy of Aromatic Molecules. J. Chem. Phys..

[72] Pople J.A. (1956). Proton Magnetic Resonance of Hydrocarbons. J. Chem. Phys..

[73] Waugh J.S., Fessenden R.W. (1957). Nuclear Resonance Spectra of Hydrocarbons: The Free Electron Model. J. Am. Chem. Soc..

[74] Waugh J., Fessendn R. (1958). Additions and Corrections: Nuclear Resonance Spectra of Hydrocarbons: The Free Electron Model. J. Am. Chem. Soc..

[75] Johnson C.E., Bovey F.A. (1958). Calculation of Nuclear Magnetic Resonance Spectra of Aromatic Hydrocarbons. J. Chem. Phys..

[76] Abraham R.J. (1981). A ring current model for the heme ring. J. Magn. Reson..

[77] Abraham R.J., Bedford G.R., Wright B. (1982). The NMR spectra of the porphyrins. 17—Metalloporphyrins as diamagnetic shift reagents, structural and specificity studies. Org. Magn. Reson.

[78] Abraham R.J., Bedford G.R., McNeillie D., Wright B. (1980). The NMR spectra of the porphyrins 16—zinc(II) meso-tetraphenylporphyrin (Zn TPP) as a diamagnetic shift reagent. A quantitative ring current model. Org. Magn. Reson.

[79] Kadish K.M., Smith K.M., Guilard R. (1999). NMR and EPR.

[80] Hunter C.A., Sanders J.K.M. (1990). The nature of.pi.-.pi. interactions. J. Am. Chem. Soc..

[81] Abraham R.J., Smith K.M. (1983). NMR spectra of porphyrins. 21. Applications of the ring-current model to porphyrin and chlorophyll aggregation. J. Am. Chem. Soc..

[82] Abraham R.J., Rowan A.E., Smith N.W., Smith K.M. (1993). NMR spectra of the porphyrins. Part 42. The synthesis and aggregation behaviour of some chlorophyll analogues. J. Chem. Soc. Perkin Trans. 2.

[83] Hynninen P.H., Lötjönen S. (1993). Effects of π−π interactions on the 1H-NMR spectra and solution structures of pheophytin a and a′ dimers. Biochim. Biophys. Acta.

[84] Vermathen M., Marzorati M., Bigler P. (2013). Self-assembling properties of porphyrinic photosensitizers and their effect on membrane interactions probed by NMR spectroscopy. J. Phys. Chem. B.

[85] Williamson M.P. (2013). Using chemical shift perturbation to characterise ligand binding. Prog. Nucl. Magn. Reson. Spectrosc..

[86] Yu Z., Li P., Merz K.M. (2017). Using Ligand-Induced Protein Chemical Shift Perturbations To Determine Protein–Ligand Structures. Biochemistry.

[87] Hunashal Y., Cantarutti C., Giorgetti S., Marchese L., Molinari H., Niccolai N., Fogolari F., Esposito G. (2020). Exploring exchange processes in proteins by paramagnetic perturbation of NMR spectra. Phys. Chem. Chem. Phys..

[88] Mayo B.C. (1973). Lanthanide shift reagents in nuclear magnetic resonance spectroscopy. Chem. Soc. Rev..

[89] von Ammon R., Fischer R.D. (1972). Shift Reagents in NMR Spectroscopy. Angew. Chem. Int. Ed..

[90] Neuhaus D., Williamson M.P. (2000). The Nuclear Overhauser Effect in Structural and Conformational Analysis.

[91] Macura S., Huang Y., Suter D., Ernst R.R. (1981). Two-dimensional chemical exchange and cross-relaxation spectroscopy of coupled nuclear spins. J. Magn. Reson..

[92] Macura S., Ernst R.R. (1980). Elucidation of cross relaxation in liquids by two-dimensional N.M.R. spectroscopy. Mol. Phys..

[93] Kumar A., Ernst R.R., Wüthrich K. (1980). A two-dimensional nuclear Overhauser enhancement (2D NOE) experiment for the elucidation of complete proton-proton cross-relaxation networks in biological macromolecules. Biochem. Biophys. Res. Commun..

[94] Butts C.P., Jones C.R., Towers E.C., Flynn J.L., Appleby L., Barron N.J. (2011). Interproton distance determinations by NOE—Surprising accuracy and precision in a rigid organic molecule. Org. Biomol. Chem..

[95] Jones C.R., Butts C.P., Harvey J.N. (2011). Accuracy in determining interproton distances using Nuclear Overhauser Effect data from a flexible molecule. Beilstein J. Org. Chem..

[96] Vögeli B., Segawa T.F., Leitz D., Sobol A., Choutko A., Trzesniak D., van Gunsteren W., Riek R. (2009). Exact Distances and Internal Dynamics of Perdeuterated Ubiquitin from NOE Buildups. J. Am. Chem. Soc..

[97] Bothner-By A.A., Stephens R.L., Lee J., Warren C.D., Jeanloz R.W. (1984). Structure determination of a tetrasaccharide: Transient nuclear Overhauser effects in the rotating frame. J. Am. Chem. Soc..

[98] Bauer C.J., Frenkiel T.A., Lane A.N. (1990). A comparison of the ROESY and NOESY experiments for large molecules, with application to nucleic acids. J. Magn. Reson..

[99] Williamson M.P., Worsfold P., Poole C., Townshend A., Miró M. (2019). Nuclear Magnetic Resonance Spectroscopy | Nuclear Overhauser Effect. Encyclopedia of Analytical Science.

[100] Stejskal E.O., Tanner J.E. (1965). Spin Diffusion Measurements: Spin Echoes in the Presence of a Time-Dependent Field Gradient. J. Chem. Phys..

[101] Cohen Y., Avram L., Frish L. (2005). Diffusion NMR Spectroscopy in Supramolecular and Combinatorial Chemistry: An Old Parameter—New Insights. Angew. Chem. Int. Ed..

[102] Johnson C.S. (1999). Diffusion ordered nuclear magnetic resonance spectroscopy: Principles and applications. Prog. Nucl. Magn. Reson. Spectrosc..

[103] Groves P. (2017). Diffusion ordered spectroscopy (DOSY) as applied to polymers. Polym. Chem..

[104] Morris K.F., Johnson C.S. (1992). Diffusion-ordered two-dimensional nuclear magnetic resonance spectroscopy. J. Am. Chem. Soc..

[105] Balayssac S., Delsuc M.-A., Gilard V., Prigent Y., Malet-Martino M. (2009). Two-dimensional DOSY experiment with Excitation Sculpting water suppression for the analysis of natural and biological media. J. Magn. Reson..

[106] Simpson A.J. (2002). Determining the molecular weight, aggregation, structures and interactions of natural organic matter using diffusion ordered spectroscopy. Magn. Reson. Chem..

[107] Colbourne A.A., Morris G.A., Nilsson M. (2011). Local Covariance Order Diffusion-Ordered Spectroscopy: A Powerful Tool for Mixture Analysis. J. Am. Chem. Soc..

[108] Pagès G., Gilard V., Martino R., Malet-Martino M. (2017). Pulsed-field gradient nuclear magnetic resonance measurements (PFG NMR) for diffusion ordered spectroscopy (DOSY) mapping. Analyst.

[109] Cohen Y., Avram L., Evan-Salem T., Frish L., Schalley C. (2006). Diffusion NMR in Supramolecular Chemistry. Analytical Methods in Supramolecular Chemistry.

[110] Kharlamov S.V., Latypov S.K. (2010). Modern diffusion-ordered NMR spectroscopy in chemistry of supramolecular systems: The scope and limitations. Russ. Chem. Rev.

[111] Bakkour Y., Darcos V., Li S., Coudane J. (2012). Diffusion ordered spectroscopy (DOSY) as a powerful tool for amphiphilic block copolymer characterization and for critical micelle concentration (CMC) determination. Polym. Chem..

[112] Khodov I.A., Alper G.A., Mamardashvili G.M., Mamardashvili N.Z. (2015). Hybrid multi-porphyrin supramolecular assemblies: Synthesis and structure elucidation by 2D DOSY NMR studies. J. Mol. Struct..

[113] Johnston M.R., Latter M.J. (2002). Characterization of porphyrin supramolecular complexes using NMR diffusion spectroscopy. J. Porphyr. Phthalocyanines.

[114] Chatzigiannis C.M., Kiriakidi S., Tzakos A.G., Mavromoustakos T., Mavromoustakos T., Tzakos A.G., Durdagi S. (2021). 2D DOSYTwo-dimensional diffusion-ordered NMR spectroscopy (2D DOSY) NMR: A Valuable Tool to Confirm the Complexation in Drug Delivery Systems. Supramolecules in Drug Discovery and Drug Delivery: Methods and Protocols.

[115] Momot K.I., Kuchel P.W. (2003). Pulsed field gradient nuclear magnetic resonance as a tool for studying drug delivery systems. Concepts Magn. Reson. Part A.

[116] Bruker Corporation (2012). Almanac—Bruker Corporation [Brochure]: Analytical Tables and Product Overview.

[117] Harris R.K. (1976). Nmr and the periodic table. Chem. Soc. Rev..

[118] Patching S.G. (2016). NMR active nuclei for biological and biomedical applications. J. Diagn. Imaging Ther..

[119] Cavanagh J., Fairbrother W.J., Palmer A.G., Rance M., Skelton N.J., Cavanagh J., Fairbrother W.J., Palmer A.G., Rance M., Skelton N.J. (2007). Chapter 7—Heteronuclear nmr experiments. Protein NMR Spectroscopy.

[120] Watts A., Roberts G.C.K. (2013). NMR of Lipids. Encyclopedia of Biophysics.

[121] Nieto L., Jiménez-Barbero J., Roberts G.C.K. (2013). Carbohydrate NMR Spectroscopy. Encyclopedia of Biophysics.

[122] Marion D. (2013). An introduction to biological NMR spectroscopy. Mol. Cell. Proteom. MCP.

[123] Lindon J.C., Worsfold P., Poole C., Townshend A., Miró M. (2016). Nuclear Magnetic Resonance Spectroscopy | Fluorine-19. Encyclopedia of Analytical Science.

[124] Goslinski T., Piskorz J. (2011). Fluorinated porphyrinoids and their biomedical applications. J. Photochem. Photobiol. C.

[125] Oz G., Pountney D.L., Armitage I.M. (1998). NMR spectroscopic studies of I = 1/2 metal ions in biological systems. Biochem Cell Biol.

[126] Moan J., Berg K., Kvam E., Western A., Malik Z., Rück A., Schneckenburger H., Bock G., Harnett S. (2007). Intracellular Localization of Photosensitizers. Ciba Foundation Symposium 146—Photosensitizing Compounds: Their Chemistry, Biology and Clinical Use.

[127] Lavi A., Weitman H., Holmes R.T., Smith K.M., Ehrenberg B. (2002). The Depth of Porphyrin in a Membrane and the Membrane’s Physical Properties Affect the Photosensitizing Efficiency. Biophys. J..

[128] Warschawski D.E., Arnold A.A., Beaugrand M., Gravel A., Chartrand É., Marcotte I. (2011). Choosing membrane mimetics for NMR structural studies of transmembrane proteins. Biochim. Biophys. Acta.

[129] Da Costa G., Mouret L., Chevance S., Le Rumeur E., Bondon A. (2007). NMR of molecules interacting with lipids in small unilamellar vesicles. Eur. Biophys. J..

[130] Da Costa G., Chevance S., Le Rumeur E., Bondon A. (2006). Proton NMR detection of porphyrins and cytochrome C in small unilamellar vesicles: Role of the dissociation kinetic constant. Biophys. J..

[131] Vermathen M., Vermathen P., Simonis U., Bigler P. (2008). Time-dependent interactions of the two porphyrinic compounds chlorin e6 and mono-L-aspartyl-chlorin e6 with phospholipid vesicles probed by NMR spectroscopy. Langmuir.

[132] Barsukov L.I., Victorov A.V., Vasilenko I.A., Evstigneeva R.P., Bergelson L.D. (1980). Investigation of the inside-outside distribution, intermembrane exchange and transbilayer movement of phospholipids in sonicated vesicles by shift reagent NMR. Biochim. Biophys. Acta.

[133] Vermathen M., Marzorati M., Vermathen P., Bigler P. (2010). pH-dependent distribution of chlorin e6 derivatives across phospholipid bilayers probed by NMR spectroscopy. Langmuir.

[134] Marzorati M., Bigler P., Vermathen M. (2011). Interactions between selected photosensitizers and model membranes: An NMR classification. Biochim. Biophys. Acta.

[135] Allen T.M., Cullis P.R. (2013). Liposomal drug delivery systems: From concept to clinical applications. Adv. Drug Deliv. Rev..

[136] Temizel E., Sagir T., Ayan E., Isik S., Ozturk R. (2014). Delivery of lipophilic porphyrin by liposome vehicles: Preparation and photodynamic therapy activity against cancer cell lines. Photodiagnosis Photodyn. Ther..

[137] Sadasivam M., Avci P., Gupta G.K., Lakshmanan S., Chandran R., Huang Y.Y., Kumar R., Hamblin M.R. (2013). Self-assembled liposomal nanoparticles in photodynamic therapy. Eur. J. Nanomed..

[138] Ikeda A., Hino S., Mae T., Tsuchiya Y., Sugikawa K., Tsukamoto M., Yasuhara K., Shigeto H., Funabashi H., Kuroda A. (2015). Porphyrin-uptake in liposomes and living cells using an exchange method with cyclodextrin. RSC Adv..

[139] Nakaya T., Tsuchiya Y., Horiguchi B., Sugikawa K., Komaguchi K., Ikeda A. (2018). 1H NMR Determination of Incorporated Porphyrin Location in Lipid Membranes of Liposomes. Bull. Chem. Soc. Jpn..

[140] Bertini I., Turano P., Vila A.J. (1993). Nuclear magnetic resonance of paramagnetic metalloproteins. Chem. Rev..

[141] Stojanović S., Isenović E.R., Zarić B.L. (2012). Non-canonical interactions of porphyrins in porphyrin-containing proteins. Amino Acids.

[142] Mitchell J., Yanamala N., Tan Y.L., Gardner E.E., Tirupula K.C., Balem F., Sheves M., Nietlispach D., Klein-Seetharaman J. (2019). Structural and Functional Consequences of the Weak Binding of Chlorin e6 to Bovine Rhodopsin. Photochem. Photobiol..

[143] Gjuroski I., Girousi E., Meyer C., Hertig D., Stojkov D., Fux M., Schnidrig N., Bucher J., Pfister S., Sauser L. (2019). Evaluation of polyvinylpyrrolidone and block copolymer micelle encapsulation of serine chlorin e6 and chlorin e4 on their reactivity towards albumin and transferrin and their cell uptake. J. Control. Release.

[144] Fiel R.J., Howard J.C., Mark E.H., Gupta N.D. (1979). Interaction of DNA with a porphyrin ligand: Evidence for intercalation. Nucleic Acids Res..

[145] Fiel R.J., Munson B.R. (1980). Binding of meso-tetra (4-N-methylpyridyl) porphine to DNA. Nucleic Acids Res..

[146] Carvlin M.J., Fiel R.J. (1983). Intercalative and nonintercalative binding of large cationic porphyrin ligands to calf thymus DNA. Nucleic Acids Res..

[147] Banville D.L., Marzilli L.G., Wilson W.D. (1983). 31P NMR and viscometric studies of the interaction of meso-tetra(4-N-methyl-pyridyl)porphine and its Ni(II) and Zn(II) derivatives with DNA. Biochem. Biophys. Res. Commun..

[148] Fiel R.J. (1989). Porphyrin—Nucleic Acid Interactions: A Review. J. Biomol. Struct. Dyn..

[149] Pratviel G. (2016). Porphyrins in complex with DNA: Modes of interaction and oxidation reactions. Coord. Chem. Rev..

[150] Wang Y., Dong Z., Hu H., Yang Q., Hou X., Wu P. (2019). DNA-modulated photosensitization: Current status and future aspects in biosensing and environmental monitoring. Anal. Bioanal. Chem..

[151] Zhao Y.-M., Lu Q.-Q., Yao S., Su H.-F., Liu H.-J., Wang Z.-J., Wu F.-S., Wang K. (2019). N-Methylpyridylporphyrin tailed with folate conjugate as a potential lysosomal-targeted photosensitizer: Synthesis, DNA interaction, singlet oxygen and subcellular localization. J. Porphyr. Phthalocyanines.

[152] Hirakawa K., Hirano T., Nishimura Y., Arai T., Nosaka Y. (2011). Control of Singlet Oxygen Generation Photosensitized by meso-Anthrylporphyrin through Interaction with DNA. Photochem. Photobiol..

[153] Hirakawa K., Harada M., Okazaki S., Nosaka Y. (2012). Controlled generation of singlet oxygen by a water-soluble meso-pyrenylporphyrin photosensitizer through interaction with DNA. Chem. Commun..

[154] Hirakawa K., Nishimura Y., Arai T., Okazaki S. (2013). Singlet Oxygen Generating Activity of an Electron Donor Connecting Porphyrin Photosensitizer Can Be Controlled by DNA. J. Phys. Chem. B.

[155] Hirakawa K., Taguchi M., Okazaki S. (2015). Relaxation Process of Photoexcited meso-Naphthylporphyrins while Interacting with DNA and Singlet Oxygen Generation. J. Phys. Chem. B.

[156] López-Cebral R., Martín-Pastor M., Seijo B., Sanchez A. (2014). Progress in the characterization of bio-functionalized nanoparticles using NMR methods and their applications as MRI contrast agents. Prog. Nucl. Magn. Reson. Spectrosc..

[157] Haaf F., Sanner A., Straub F. (1985). Polymers of N-Vinylpyrrolidone: Synthesis, Characterization and Uses. Polym. J..

[158] Luo Y., Hong Y., Shen L., Wu F., Lin X. (2021). Multifunctional Role of Polyvinylpyrrolidone in Pharmaceutical Formulations. AAPS PharmSciTech.

[159] Franco P., De Marco I. (2020). The Use of Poly(N-vinyl pyrrolidone) in the Delivery of Drugs: A Review. Polymers.

[160] Copley L., van der Watt P., Wirtz K.W., Parker M.I., Leaner V.D. (2008). Photolon™, a chlorin e6 derivative, triggers ROS production and light-dependent cell death via necrosis. Int. J. Biochem. Cell Biol..

[161] Zhiyentayev T.M., Boltaev U.T., Solov’eva A.B., Aksenova N.A., Glagolev N.N., Chernjak A.V., Melik-Nubarov N.S. (2014). Complexes of Chlorin e6 with Pluronics and Polyvinylpyrrolidone: Structure and Photodynamic Activity in Cell Culture. Photochem. Photobiol..

[162] Tsvetkov V.B., Solov’eva A.B., Melik-Nubarov N.S. (2014). Computer modeling of the complexes of Chlorin e6 with amphiphilic polymers. Phys. Chem. Chem. Phys..

[163] Hädener M., Gjuroski I., Furrer J., Vermathen M. (2015). Interactions of Polyvinylpyrrolidone with Chlorin e6-Based Photosensitizers Studied by NMR and Electronic Absorption Spectroscopy. J. Phys. Chem. B.

[164] Solov’eva A.B., Khasanova O.V., Aksenova N.A., Chernyak A.V., Volkov V.I., Timofeeva V.A., Timashev P.S. (2019). The Influence of Effect of Polysaccharides and Polyvinylpyrrolidone on the Photocatalytic Activity of Chlorin e6 in Tryptophan Oxidation. Russ. J. Phys. Chem. A.

[165] Gjuroski I., Furrer J., Vermathen M. (2018). How Does the Encapsulation of Porphyrinic Photosensitizers into Polymer Matrices Affect Their Self-Association and Dynamic Properties?. Chemphyschem.

[166] Aksenova N.A., Oles T., Sarna T., Glagolev N.N., Chernjak A.V., Volkov V.I., Kotova S.L., Melik-Nubarov N.S., Solovieva A.B. (2012). Development of novel formulations for photodynamic therapy on the basis of amphiphilic polymers and porphyrin photosensitizers. Porphyrin-polymer complexes in model photosensitized processes. Laser Phys..

[167] Solovieva A.B., Aksenova N.A., Berlin A.A., Rogovina S.Z., Zaikov G.E. (2015). Porphyrin-Polymer Complexes in Model Photosensitized Processes and in Photodynamic Therapy. Additives in Polymers.

[168] Küçük T., Alpugan S., Davarcı D., Pehlivan E.G., Bayır S., Tazebay U.H., Dumoulin F. (2019). Photoproperties, PVP formulation and 19F NMR of a Zn phthalocyanine with 24 magnetically pseudo-equivalent fluorine atoms. J. Porphyr. Phthalocyanines.

[169] Schneider H.-J., Hacket F., Rüdiger V., Ikeda H. (1998). NMR Studies of Cyclodextrins and Cyclodextrin Complexes. Chem. Rev..

[170] Gonzalez M.C., Weedon A.C. (1985). Preparation and properties of a linked porphyrin–cyclodextrin. Can. J. Chem..

[171] Kuroda Y., Hiroshige T., Sera T., Shiroiwa Y., Tanaka H., Ogoshi H. (1989). Cyclodextrin-sandwiched porphyrin. J. Am. Chem. Soc..

[172] Dick D.L., Rao T.V.S., Sukumaran D., Lawrence D.S. (1992). Molecular encapsulation: Cyclodextrin-based analogs of heme-containing proteins. J. Am. Chem. Soc..

[173] Kano K., Kitagishi H., Dagallier C., Kodera M., Matsuo T., Hayashi T., Hisaeda Y., Hirota S. (2006). Iron Porphyrin−Cyclodextrin Supramolecular Complex as a Functional Model of Myoglobin in Aqueous Solution. Inorg. Chem..

[174] Carofiglio T., Fornasier R., Lucchini V., Rosso C., Tonellato U. (1996). Very strong binding and mode of complexation of water-soluble porphyrins with a permethylated β-cyclodextrin. Tetrahedron Lett..

[175] Kadish K.M., Maiya G.B., Araullo C., Guilard R. (1989). Micellar effects on the aggregation of tetraanionic porphyrins. Spectroscopic characterization of free-base meso-tetrakis(4-sulfonatophenyl)porphyrin, (TPPS)H2, and (TPPS)M (M = zinc(II), copper(II), and vanadyl) in aqueous micellar media. Inorg. Chem..

[176] Ribó J.M., Farrera J.-A., Valero M.L., Virgili A. (1995). Self-assembly of cyclodextrins with meso-tetrakis(4-sulfonatophenyl)porphyrin in aqueous solution. Tetrahedron.

[177] Sur S.K., Bryant R.G. (1995). Spin-Lattice Relaxation Enhancement of Water Protons by Manganese Porphyrins Complexed with Cyclodextrins. J. Phys. Chem..

[178] Mosseri S., Mialocq J.C., Perly B., Hambright P. (1991). Porhyrins-cyclodextrin 1 Photooxidation of zinc tetrakis(4-sulfonatophenyl)porphyrin in cyclodextrin cavities: The characterization of ZnTSPP dication Photolysis, radiolysis, and NMR studies. J. Phys. Chem..

[179] Mosinger J., Kliment V., Sejbal J., Kubát P., Lang K. (2002). Host-guest complexes of anionic porphyrin sensitizers with cyclodextrins. J. Porphyr. Phthalocyanines.

[180] Kano K., Nishiyabu R., Asada T., Kuroda Y. (2002). Static and Dynamic Behavior of 2:1 Inclusion Complexes of Cyclodextrins and Charged Porphyrins in Aqueous Organic Media. J. Am. Chem. Soc..

[181] Ma H.L., Wu J.J., Liang W.J., Chao J.B. (2007). Study on the Association Phenomenon of Cyclodextrin to Porphyrin J-aggregates by NMR Spectroscopy. J. Incl. Phenom. Macrocycl. Chem..

[182] El-Hachemi Z., Farrera J.-A., Garcia-Ortega H., Ramirez-Gutierrez O., Ribo J.M. (2001). Heteroassociation of meso-sulfonatophenylporphyrins with β- and γ-cyclodextrin. J. Porphyr. Phthalocyanines.

[183] Venema F., Rowan A.E., Nolte R.J.M. (1996). Binding of Porphyrins in Cyclodextrin Dimers. J. Am. Chem. Soc..

[184] Mosinger J., Slavětínská L., Lang K., Coufal P., Kubát P. (2009). Cyclodextrin carriers of positively charged porphyrin sensitizers. Org. Biomol. Chem..

[185] Xiliang G., Shaomin S., Chuan D., Feng F., Wong M.S. (2005). Comparative study on the inclusion behavior between meso-tetrakis(4-N-ethylpyridiniurmyl)porphyrin and β-cyclodextrin derivatives. Spectrochim. Acta A.

[186] Khurana R., Kakatkar A.S., Chatterjee S., Barooah N., Kunwar A., Bhasikuttan A.C., Mohanty J. (2019). Supramolecular Nanorods of (N-Methylpyridyl) Porphyrin With Captisol: Effective Photosensitizer for Anti-bacterial and Anti-tumor Activities. Front. Chem..

[187] Guo Y.-J., Chao J.-B., Pan J.-H. (2007). Study on the interaction of 5-pyridine-10,15,20-tris-(p-chlorophenyl)porphyrin with cyclodextrins and DNA by spectroscopy. Spectrochim. Acta A.

[188] Cosma P., Catucci L., Fini P., Dentuto P.L., Agostiano A., Angelini N., Scolaro L.M. (2006). Tetrakis(4-pyridyl)porphyrin supramolecular complexes with cyclodextrins in aqueous solution. Photochem. Photobiol..

[189] Notsu S., Sugikawa K., Ikeda A. (2018). Reversible Supramolecular System of Porphyrin Exchange between Inclusion in Cyclodextrin and Intercalation in DNA by Change in pH. ChemistrySelect.

[190] Ikeda A., Satake S., Mae T., Ueda M., Sugikawa K., Shigeto H., Funabashi H., Kuroda A. (2017). Photodynamic Activities of Porphyrin Derivative–Cyclodextrin Complexes by Photoirradiation. ACS Med. Chem. Lett..

[191] Kitagishi H., Hatada S., Itakura T., Maki Y., Maeda Y., Kano K. (2013). Cellular uptake of octaarginine-conjugated tetraarylporphyrin included by per-O-methylated β-cyclodextrin. Org. Biomol. Chem..

[192] Králová J., Kejík Z., Bříza T., Poučková P., Král A., Martásek P., Král V. (2010). Porphyrin−Cyclodextrin Conjugates as a Nanosystem for Versatile Drug Delivery and Multimodal Cancer Therapy. J. Med. Chem..

[193] Puglisi A., Purrello R., Rizzarelli E., Sortino S., Vecchio G. (2007). Spectroscopic and self-association behavior of a porphyrin-β-cyclodextrin conjugate. New J. Chem..

[194] Mineo P. (2014). A porphyrin/β-cyclodextrin conjugated nano-system having a pan–lid molecular structure for smart drug carrier applications. Org. Biomol. Chem..

[195] Zhao J., Zhang H.Y., Sun H.L., Liu Y. (2015). Supramolecular nanoassemblies of an amphiphilic porphyrin-cyclodextrin conjugate and their morphological transition from vesicle to network. Chemistry.

[196] Sun M., Zhang H.-Y., Liu B.-W., Liu Y. (2013). Construction of a Supramolecular Polymer by Bridged Bis(permethyl-β-cyclodextrin)s with Porphyrins and Its Highly Efficient Magnetic Resonance Imaging. Macromolecules.

[197] Liu Y., Ke C.-F., Zhang H.-Y., Cui J., Ding F. (2008). Complexation-Induced Transition of Nanorod to Network Aggregates:  Alternate Porphyrin and Cyclodextrin Arrays. J. Am. Chem. Soc..

[198] Zhang H., Zhang B., Zhu M., Grayson S.M., Schmehl R., Jayawickramarajah J. (2014). Water-soluble porphyrin nanospheres: Enhanced photo-physical properties achieved via cyclodextrin driven double self-inclusion. Chem. Commun..

[199] Cerichelli G., Mancinit G. (1997). NMR techniques applied to micellar systems. Curr. Opin. Colloid Interface Sci..

[200] Söderman O., Stilbs P., Price W.S. (2004). NMR studies of surfactants. Concepts in Magnetic Resonance Part A.

[201] Cui X., Mao S., Liu M., Yuan H., Du Y. (2008). Mechanism of Surfactant Micelle Formation. Langmuir.

[202] Medhi O.K., Mazumdar S., Mitra S. (1989). Proton NMR and optical spectra and magnetic properties of four-coordinated intermediate-spin, five-coordinated high-spin, and six-coordinated low-spin iron(II) hemes encapsulated in aqueous detergent micelles: Model for hemoproteins. Inorg. Chem..

[203] Mazumdar S. (1990). Proton and carbon-13 NMR studies on the structure of micelles encapsulating hemes in aqueous sodium dodecyl sulfate solutions. J. Phys. Chem..

[204] Mazumdar S., Medhi O.K., Mitra S. (1991). Stability and characterization of iron(III) and iron(II) heme peptides encapsulated in aqueous detergent micelles: Proton NMR and UV-visible spectroscopic studies. Inorg. Chem..

[205] Mazumdar S., Mitra S. (1993). Biomimetic chemistry of hemes inside aqueous micelles. Structures and Biological Effects.

[206] Maiti N.C., Mazumdar S., Periasamy N. (1995). Dynamics of Porphyrin Molecules in Micelles. Picosecond Time-Resolved Fluorescence Anisotropy Studies. J. Phys. Chem..

[207] Gandini S.C.M., Yushmanov V.E., Borissevitch I.E., Tabak M. (1999). Interaction of the Tetra(4-sulfonatophenyl)porphyrin with Ionic Surfactants:  Aggregation and Location in Micelles. Langmuir.

[208] Yushmanov V.E. (1999). Aggregation of Fe(III)TPPS4 on Biological Structures Is pH-Dependent, Suggesting Oxo-Bridging in the Aggregates. Inorg. Chem..

[209] Kadish K.M., Maiya B.G., Araullo-McAdams C. (1991). Spectroscopic characterization of meso-tetrakis(1-methylpyridinium-4-yl)porphyrins, [(TMpyP)H2]4+ and [(TMpyP)M]4+, in aqueous micellar media, where M = VO2+, Cu(II), and Zn(II). J. Phys. Chem..

[210] Monsú Scolaro L., Donato C., Castriciano M., Romeo A., Romeo R. (2000). Micellar aggregates of platinum(II) complexes containing porphyrins. Inorg. Chim. Acta.

[211] Vermathen M., Louie E.A., Chodosh A.B., Ried S., Simonis U. (2000). Interactions of Water-Insoluble Tetraphenylporphyrins with Micelles Probed by UV−Visible and NMR Spectroscopy. Langmuir.

[212] Maiti N.C., Mazumdar S., Periasamy N. (1998). J- and H-Aggregates of Porphyrin−Surfactant Complexes:  Time-Resolved Fluorescence and Other Spectroscopic Studies. J. Phys. Chem. B.

[213] Qiu W.-G., Li Z.-F., Bai G.-M., Meng S.-N., Dai H.-X., He H. (2007). Interaction of water-soluble cationic porphyrin with anionic surfactant. Spectrochim. Acta A.

[214] Gandini S.C.M., Yushmanov V.E., Tabak M. (2001). Interaction of Fe(III)- and Zn(II)-tetra(4-sulfonatophenyl) porphyrins with ionic and nonionic surfactants: Aggregation and binding. J. Inorg. Biochem..

[215] Mamardashvili G.M., Kaigorodova E.Y., Khodov I.y.A., Scheblykin I., Mamardashvili N.Z., Koifman O.I. (2019). Micelles encapsulated Co(III)-tetra(4-sulfophenyl)porphyrin in aqueous CTAB solutions: Micelle formation, imidazole binding and redox Co(III)/Co(II) processes. J. Mol. Liq..

[216] Mamardashvili G.M., Kaigorodova E.Y., Simonova O.R., Lazovskiy D.A., Mamardashvili N.Z. (2020). Interaction of the Sn(IV)-tetra(4-sulfonatophenyl)porphyrin axial complexes with cetyltrimethylammonium bromide: Aggregation and location in micelles, fluorescence properties and photochemical stability. J. Mol. Liq..

[217] Cabral H., Miyata K., Osada K., Kataoka K. (2018). Block Copolymer Micelles in Nanomedicine Applications. Chem. Rev..

[218] Biswas S., Kumari P., Lakhani P.M., Ghosh B. (2016). Recent advances in polymeric micelles for anti-cancer drug delivery. Eur. J. Pharm. Sci..

[219] Sakai-Kato K., Nishiyama N., Kozaki M., Nakanishi T., Matsuda Y., Hirano M., Hanada H., Hisada S., Onodera H., Harashima H. (2015). General considerations regarding the in vitro and in vivo properties of block copolymer micelle products and their evaluation. J. Control. Release.

[220] Houdaihed L., Evans J.C., Allen C. (2017). Overcoming the Road Blocks: Advancement of Block Copolymer Micelles for Cancer Therapy in the Clinic. Mol. Pharm..

[221] Fraenza C.C., Mattea C., Farrher G.D., Ordikhani-Seyedlar A., Stapf S., Anoardo E. (2018). Rouse dynamics in PEO-PPO-PEO block-copolymers in aqueous solution as observed through fast field-cycling NMR relaxometry. Polymer.

[222] Walderhaug H., Söderman O. (2009). NMR studies of block copolymer micelles. Curr. Opin. Colloid Interface Sci..

[223] Ma J.-H., Guo C., Tang Y.-L., Liu H.-Z. (2007). 1H NMR Spectroscopic Investigations on the Micellization and Gelation of PEO−PPO−PEO Block Copolymers in Aqueous Solutions. Langmuir.

[224] Ma J.-H., Guo C., Tang Y.-L., Wang J., Zheng L., Liang X.-F., Chen S., Liu H.-Z. (2007). Salt-Induced Micellization of a Triblock Copolymer in Aqueous Solution:  A 1H Nuclear Magnetic Resonance Spectroscopy Study. Langmuir.

[225] Nilsson M., Håkansson B., Söderman O., Topgaard D. (2007). Influence of Polydispersity on the Micellization of Triblock Copolymers Investigated by Pulsed Field Gradient Nuclear Magnetic Resonance. Macromolecules.

[226] Pfister S., Sauser L., Gjuroski I., Furrer J., Vermathen M. (2019). Monitoring the encapsulation of chlorin e6 derivatives into polymer carriers by NMR spectroscopy. J. Porphyr. Phthalocyanines.

[227] Izunobi J.U., Higginbotham C.L. (2011). Polymer Molecular Weight Analysis by 1H NMR Spectroscopy. J. Chem. Educ..

[228] Batrakova E.V., Kabanov A.V. (2008). Pluronic block copolymers: Evolution of drug delivery concept from inert nanocarriers to biological response modifiers. J. Control. Release.

[229] Cacaccio J., Durrani F., Cheruku R.R., Borah B., Ethirajan M., Tabaczynski W., Pera P., Missert J.R., Pandey R.K. (2020). Pluronic F-127: An Efficient Delivery Vehicle for 3-(1’-hexyloxy)ethyl-3-devinylpyropheophorbide-a (HPPH or Photochlor). Photochem. Photobiol..

[230] Mike Motloung B., Edward Sekhosana K., Managa M., Prinsloo E., Nyokong T. (2019). The photophysicochemical properties and photodynamic therapy activity of phenyldiazenyl phenoxy substituted phthalocyanines when incorporated into Pluronic^®^ F127 micelles. Polyhedron.

[231] Wenceslau A.C., Ferreira G.L.Q.C., Hioka N., Caetano W. (2015). Spectroscopic studies of pyridil and methoxyphenyl porphyrins in homogeneous and Pluronic^®^-based nanostructured systems. J. Porphyr. Phthalocyanines.

[232] D’Souza A.A., Shegokar R. (2016). Polyethylene glycol (PEG): A versatile polymer for pharmaceutical applications. Expert Opin. Drug Deliv..

[233] Zalipsky S. (1995). Chemistry of polyethylene glycol conjugates with biologically active molecules. Adv. Drug Del. Rev..

[234] Harris J.M., Martin N.E., Modi M. (2001). Pegylation: A novel process for modifying pharmacokinetics. Clin. Pharmacokinet..

[235] Swierczewska M., Lee K.C., Lee S. (2015). What is the future of PEGylated therapies?. Expert. Opin. Emerg. Drugs.

[236] Steinbeck C.A., Hedin N., Chmelka B.F. (2004). Interactions of Charged Porphyrins with Nonionic Triblock Copolymer Hosts in Aqueous Solutions. Langmuir.

[237] Lamch Ł., Tylus W., Jewgiński M., Latajka R., Wilk K.A. (2016). Location of Varying Hydrophobicity Zinc(II) Phthalocyanine-Type Photosensitizers in Methoxy Poly(ethylene oxide) and Poly(l-lactide) Block Copolymer Micelles Using 1H NMR and XPS Techniques. J. Phys. Chem. B.

[238] Lepre C.A., Moore J.M., Peng J.W. (2004). Theory and applications of NMR-based screening in pharmaceutical research. Chem. Rev..

[239] Bloembergen N., Purcell E.M., Pound R.V. (1948). Relaxation Effects in Nuclear Magnetic Resonance Absorption. Phys. Rev..

